# Natural Gastrointestinal Stable Pea Albumin Nanomicelles for Capsaicin Delivery and Their Effects for Enhanced Mucus Permeability at Small Intestine

**DOI:** 10.34133/bmr.0065

**Published:** 2024-08-16

**Authors:** Yiming Li, Mengqi Mao, Xin Yuan, Jiajia Zhao, Lingjun Ma, Fang Chen, Xiaojun Liao, Xiaosong Hu, Junfu Ji

**Affiliations:** College of Food Science and Nutritional Engineering, National Engineering Research Center for Fruit and Vegetable Processing, China Agricultural University, Key Lab of Fruit and Vegetable Processing, Ministry of Agriculture and Rural Affairs, Beijing 100083, China.

## Abstract

Natural nanodelivery systems are highly desirable owing to their biocompatibility and biodegradability. However, these delivery systems face challenges from potential degradation in the harsh gastrointestinal environment and limitations imposed by the intestinal mucus barrier, reducing their oral delivery efficacy. Here, gastrointestinal stable and mucus-permeable pea albumin nanomicelles (PANs) with a small particle size (36.42 nm) are successfully fabricated via pre-enzymatic hydrolysis of pea albumin isolate (PAI) using trypsin. Capsaicin (CAP) is used as a hydrophobic drug model and loaded in PAN with a loading capacity of 20.02 μg/mg. PAN exhibits superior intestinal stability, with a 40% higher CAP retention compared to PAI in simulated intestinal digestion. Moreover, PAN displays unrestricted movement in intestinal mucus and can effectively penetrate it, since it increases the mucus permeability of CAP by 2.5 times, indicating an excellent ability to overcome the mucus barrier. Additionally, PAN enhances the cellular uptake and transcellular transport of CAP with endoplasmic reticulum/Golgi and Golgi/plasma membrane pathways involved in the transcytosis and exocytosis. This study suggests that partially enzymatically formed PAN may be a promising oral drug delivery system, effectively overcoming the harsh gastrointestinal environment and mucus barrier to improve intestinal absorption and bioavailability of hydrophobic bioactive substances.

## Introduction

Currently, nanodrug delivery systems have been extensively designed to enhance the stability and bioavailability of sensitive bioactive compounds. Commonly employed materials for constructing these delivery systems include synthetic polymers as well as natural polymers such as proteins, polysaccharides, and lipids [1,2]. Among them, synthetic polymers are limited in application due to their cytotoxicity or lack of biocompatibility and biodegradability, while natural polymers emerge as more advantageous alternatives in overcoming these drawbacks [3,4]. However, there are still some aspects of using natural polymers to build delivery systems that deserve further improvement. First, natural polymers are subject to degradation by diverse digestive enzymes existing in the gastrointestinal tract, which makes the nanocarriers prepared from them less stable and prone to rapid release of bioactives in the gastrointestinal tract [5,6]. Second, most nanocarriers, especially those derived from polysaccharides, exhibited a particle size exceeding 200 nm, which was larger than the pore size of the mucus covering the intestinal surface (10 to 200 nm) [7–10]. This rendered the nanocarriers susceptible to spatial hindrance by the mucus layer, thereby reducing the mucus permeability, which detrimentally influenced their subsequent internalization by intestinal epithelial cells [11,12]. Hence, to further enhance the absorption and utilization of sensitive bioactives in the intestinal tract, employing nanocarriers characterized by excellent intestinal stability and mucus permeability for their delivery would represent a more advantageous option.

Pea albumin isolate (PAI), an emerging plant protein, is an ideal candidate for the construction of nanocarriers with good intestinal stability and intestinal mucus permeability. PAI is a water-soluble globular protein, accounting for 18% to 25% of total protein in pea seeds, mainly composed of pea albumin 1 (PA1), pea albumin 2 (PA2), lipoxygenase, and lectin [13,14]. PA1 consists of PA1a (molecular weight of 6 kDa, 53 amino acids) and PA1b (molecular weight of 4 kDa, 37 amino acids), contributing about 50% of the seed’s sulfur amino acids. PA2, the main component of PAI, is composed of 2 polypeptides (PA2a and PA2b) with a molecular weight of about 24 to 25 kDa [14]. Each polypeptide chain of PA2 contains about 229 amino acids, including 3 cysteines, which account for 16% of the seed’s sulfur amino acids [15]. PAI is characterized by high sulfur content, low allergenicity, small molecular weight, and a nutritionally balanced amino acid composition [16]. Meantime, PAI has amphiphilic properties and its flexible polypeptide chains can easily change conformation, making it very suitable for constructing delivery carriers for the encapsulation and delivery of different bioactive substances [17]. Furthermore, PAI exhibits several merits compared to other legume proteins. First, it possesses a simplistic peptide chain structure with better solubility. Second, it contains anti-digestive peptides that effectively diminish protein digestibility by inhibiting protein hydrolysis, thus reaching the intestine in large quantities in an active form and facilitating the preservation and utilization of bioactive compounds [18,19]. Additionally, PAI has the advantage of easy control of particle size during the construction of nanocarriers. Given the close correlation between the particle size of nanocarriers and their mucus permeability, PAI is expected to fabricate nanocarriers with exceptional intestinal mucus permeability. While considering the use of PAI to fabricate nanocarriers with superior intestinal stability and mucus permeability, enzymatic hydrolysis may be an effective strategy.

Enzymatic hydrolysis is a method that has shown noteworthy efficacy in improving protein solubility and exposing internal hydrophobic binding sites, with the advantages of specific cleavage, easy-to-control process, and scaled-up production [20,21]. Amphiphilic peptides produced during the enzymatic hydrolysis of proteins can self-assemble into various well-defined nanostructures that exhibit excellent intestinal permeability, enabling deeper penetration through the mucus barrier and improved cellular uptake [22,23]. There are many types of enzymes used in the enzymatic hydrolysis of protein, such as alcalase, neutrase, flavorzyme, and protamex [24–26]. Compared to these enzymes, selecting human small intestinal digestive enzymes for the enzymatic hydrolysis of PAI has more potential to fabricate nanocarriers with high intestinal stability. This potential relies on the fact that pre-enzymatic hydrolysis of PAI using digestive enzymes can reduce cleavage sites, which facilitates the obtained nanocarriers to exhibit a certain level of resistance against digestive enzyme-induced degradation during intestinal digestion.

In this study, the intestinal stable amphiphilic peptides were first found and prepared. Capsaicin (CAP) was used as a hydrophobic drug model. The nanomicelles with high loading capacity were self-assembled from these natural peptides by the pH-shifting method. The intestinal stability was evaluated by in vitro simulated digestion, and the mucus permeability of nanomicelles was assessed on the Transwell permeability support system. Meanwhile, their motion behavior and three-dimensional (3D) distribution in mucus were further investigated by multiple particle tracking (MPT) technique and confocal laser scanning microscopy (CLSM), respectively. The cellular uptake, intracellular transport, and transcellular transport of nanomicelles were also investigated based on the Caco-2 cell layer model. Furthermore, a combined assessment of mucus and intestinal epithelial permeability of nanomicelles was conducted by using different segments of the rat intestine. This work showcased the potential of partially enzymatically formed pea albumin nanomicelles (PANs) as a promising delivery system for enhancing intestinal absorption and bioavailability of hydrophobic bioactive substances through overcoming the harsh gastrointestinal environment and mucus barrier.

## Materials and Methods

### Materials

Peas were purchased from Dongfangliang Life Science and Technology Co. Ltd. (Shanxi, China). CAP (98% purity) was purchased from DaXingAnLing Lingonberry Boreal Biotech Co. Ltd. (Heilongjiang, China). Trypsin from porcine pancreas (250 U/mg protein) was purchased from Shanghai Yuanye Biotechnology Co. Ltd. (Shanghai, China). Trypsin inhibitor from soybean (3,000 N-benzoyl-L-arginine-ethyl ester (BAEE) units/mg protein), phosphate-buffered solution (PBS) buffer (0.01 M, pH 7.2 to 7.4), Krebs–Ringer buffer, Dulbecco’s modified Eagle’s medium (DMEM), non-essential amino acid, cell counting kit-8 (cck-8), 4% paraformaldehyde, chlorpromazine, indomethacin, colchicine, cell tissue lysate buffer, and Hanks’ balanced salt solution (HBSS) were obtained from Solarbio Science & Technology Co. Ltd. (Beijing, China). Pepsin from porcine gastric mucosa (2,500 U/mg protein), bile salt, pancreatin from porcine pancreas (8 × the standard USP unit), 4′,6-diamidino-2-phenylindole (DAPI), sodium azide, brefeldin A, monensin, and bafilomycin A1 were purchased from Sigma-Aldrich Chemical Co. (St. Louis, MO, USA). Fetal bovine serum (FBS) was purchased from Gibco (Carlsbad, CA). Acetonitrile and Alexa Fluor 555–wheat germ agglutinin (WGA) dye were purchased from Fisher Scientific (New Jersey, USA). Fluorescein isothiocyanate (FITC) was purchased from Beijing Kulaibo Technology Co. Ltd. Phalloidin–iFluor 488 dye was purchased from Abcam (Cambridge, UK). Potassium bromide, o-phthalaldehyde (OPA), and Nile red (NR) were purchased from Aladdin Reagent Co. Ltd. (Shanghai, China). All other chemicals used were of analytical grade. The human intestinal Caco-2 cells were purchased from Peking Union Medical College (Beijing, China).

### Preparation and morphology observation of PAN

The extraction protocol of PAI was referenced from Rubio et al. [27] with slight modifications. The purity of PAI exceeded 88% measured by Kjeldahl method. PAI (0.5%, w/v) was dispersed in ultrapure water and fully hydrated overnight at 4 °C. After centrifugation (4,500*g*, 10 min, 4 °C), the supernatant was adjusted to pH 8.0 and trypsin (1%, w/w) was added to enzymatic hydrolysis for 90 min at 37 °C. Trypsin inhibitor (trypsin inhibitor: trypsin = 1:1, w/w) was added to terminate the protein hydrolysis process to obtain peptide solution. Subsequently, the peptide solution was adjusted to pH 12.0 at 25 °C, equilibrated for 30 min, and shifted back to pH 8.0, which was then passed through a 0.22-μm aqueous filter membrane to obtain the self-assembly PAN. The obtained PAN solution was lyophilized and stored at −20 °C until use. The yield of PAN was 24.60 ± 1.06%. The morphology of PAN was characterized by a transmission electron microscope (TEM) (JEM-1200EX, JEOL Ltd., Tokyo, Japan). A drop of PAN solution (100 μg/ml) was deposited on a carbon-coated copper grid and imaged under an accelerating voltage of 100 kV.

### Degree of hydrolysis of peptide solution

Degree of hydrolysis (DH) of peptide solution was determined by the OPA method [28]. Briefly, 150 μl of peptide solution was mixed with 3 ml of OPA reagent and incubated for 3 min at 25 °C. The absorbance values at 340 nm were then determined by an ultraviolet/visible (UV/VIS) spectrophotometer (UVmini-1240, Shimadzu Corp., Jiangsu, China), and the DH was calculated based on Eq. 1.

$$\text{DH}\;(\;\%\;)=\frac{h}{h_{\text{tot}}}\times 100$$ (1)

where *h* is the number of peptide bonds being hydrolyzed and *h*_tot_ is the total number of protein peptide bonds. The *h*_tot_ value in this study was 7.55. The DH determined by the OPA method was about 5.72%.

### The measurement of critical micelle concentration value

The measurement of critical micelle concentration (CMC) followed the protocol of Mallick et al. [29] with slight modifications. The NR ethanol solution was sequentially added to a range of concentrations of peptide solutions and PAI solutions (0.001 to 5 mg/ml) to its final concentration of 1 μМ. After mixing well, fluorescence intensity was scanned at 25 °C using a fluorescence spectrophotometer (F-7000, Hitachi, Tokyo, Japan). The excitation wavelength was 550 nm, the emission wavelength was 575 to 800 nm, and both slit width was 10 nm. The fluorescence intensity at 650 nm was analyzed, and the CMC value was obtained by plotting.

### The surface tension and dynamic interfacial tension of peptide solution and PAI

The surface tension and dynamic interfacial tension of peptide solution were measured using a contact angle meter (OCA25, Dataphysics, Stuttgart, Germany) according to the current study with slight modifications [30]. Various concentrations (0.01, 0.1, 1, 3, and 5 mg/ml) of peptide solutions and PAI solutions were prepared for surface tension measurement. Furthermore, oil–water interfacial tension was measured in real time for peptide solutions and PAI solutions at 0.5 and 5 mg/ml over an 1,800-s period.

### Secondary structure of PAI, peptide solution, and PAN

The secondary structure of PAI, peptide solution, and PAN were measured using a circular dichroism spectrometer (Chirascan Plus, Applied Photophysics Ltd., England, UK). Samples (0.2 mg/ml) were dispersed in ultrapure water. Spectra ranging from 195 to 260 nm were recorded using 1-mm quartz cuvettes at a scan speed of 120 nm/min and a bandwidth of 1 nm. The CDNN software was used to calculate the percentage of α-helix, β-sheet, β-turn, and random coil.

### The analysis of PAN by size exclusion chromatography and sodium dodecyl sulfate–polyacrylamide gel electrophoresis

PAN was separated and purified on a size exclusion column (TSKgel G2000SW, TOSOH, Tokyo, Japan). The flow rate was set at 3 ml/min, and the absorption wavelength was 280 nm. Samples corresponding to the specific peaks were collected and then analyzed by sodium dodecyl sulfate–polyacrylamide gel electrophoresis (SDS-PAGE). Briefly, the collected samples were mixed with loading buffer at a volume ratio of 4:1 to reach a final protein concentration of 2 mg/ml. The mixture was then boiled at 100 °C for 5 min. After cooling to 25 °C, 20 μl of mixture was added to a pre-prepared gel consisting of 5% acrylamide stacking gel and 4 to 20% gradient acrylamide separating gel. The stacking gel and separating gel were run at a constant voltage of 80 and 100 V, respectively. After electrophoresis, the gel was stained with Coomassie brilliant blue solution and then decolorized with ultrapure water. Finally, a multi-function imager (JY04S-3C, Beijing Junyi Oriental Electrophoresis Equipment Company, Beijing, China) was used for imaging analysis.

### Peptide sequence determination by liquid chromatography–mass spectrometry

The samples corresponding to the specific peaks collected during the size exclusion chromatography (SEC) were lyophilized, then redissolved in solvent A (0.1% formic acid in water), and analyzed by Orbitrap Fusion coupled to a system (EASY-nanoLC 1200, Thermo Fisher Scientific, MA, USA). Sample solutions (3 μl) were loaded onto a C18 column (Acclaim PepMap, Thermo Fisher Scientific, MA, USA) and separated with 60-min gradient starting at 5% buffer B (80% acetonitrile with 0.1% formic acid) followed by a stepwise increase to 80% in 55 min and 100% in 1 min and remained there for 4 min. The column flow rate was maintained at 400 nl/min with a column temperature of 40 °C. The electrospray voltage was set to 2 kV.

### Fabrication of CAP-loaded PAI (PAI-CAP) and CAP-loaded PAN (PAN-CAP)

PAI and peptide solution was adjusted to pH 12.0 and equilibrated for 30 min. To achieve better dispersion of CAP in water, CAP was first completely dissolved in anhydrous ethanol and then mixed with an equal amount of pH 12.0 water [31]. Anhydrous ethanol was removed by nitrogen blowing to avoid the possible effect on the denaturation of PAI and peptide. The CAP solution was slowly injected into the PAI and peptide solution (PAI/peptide solution: CAP = 10:1, w/w; PAI/peptide solution: CAP solution = 60:1, v/v). After stirring for 3 min, the pH value was shifted back to 8.0 and the unembedded CAP was removed by passing through a 0.22-μm aqueous filter membrane to obtain PAI-CAP and PAN-CAP, respectively. The obtained PAI-CAP and PAN-CAP solutions were then lyophilized and stored at −20 °C until use.

The PAI-CAP and PAN-CAP lyophilized powders (10 mg) were dispersed in 2 ml of acetonitrile followed by centrifugation (8,000*g*, 5 min). The CAP in the supernatant was considered as free CAP. Additionally, 10 mg of powders was dissolved in 0.2 ml of ultrapure water and then mixed with 1.8 ml of acetonitrile. The mixture was sonicated for 5 min followed by centrifugation (8,000*g*, 5 min). The CAP in the supernatant was considered as total CAP. The CAP content was quantified by high-performance liquid chromatography (HPLC; RF-10AXL, Shimadzu Corp., Kyoto, Japan) using a C18 column (Inertsil ODS-SP, GL Sciences, Kyoto, Japan) (5 μm, 4.6 × 150 mm) with a detection wavelength of 280 nm and an injection volume of 20 μl. The mobile phase was acetonitrile and water (85:15, v/v) with isocratic elution at a flow rate of 1.0 ml/min. The encapsulation efficiency (EE) and loading amount (LA) values were calculated according to Eqs. 2 and 3.

$$\text{EE}(\;\%\;)=\frac{\text{Total amount of}\;CAP-\text{free}\;CAP}{\text{Total amount of}\;CAP}$$ (2)

$$\text{LA}(\;\%\;)=\frac{\text{Total amount of}\;CAP-\text{free}\;CAP}{\text{Total weight of nanomicelles}}$$ (3)

### Interactions between CAP and peptides

The samples (2 mg) and dried potassium bromide (198 mg) were mixed and ground, which were measured by a Fourier transform infrared (FTIR) spectrometer (Tensor 27, Bruker, Karlsruhe, Germany) from 400 to 4,000 cm^−1^ with a resolution of 4 cm^−1^. In addition, the interactions were measured by the fluorescence spectrophotometer (F-7000, Hitachi, Tokyo, Japan). Sample solutions (0.5 mg/ml) were prepared using ultrapure water. Emission spectra were recorded from 300 to 450 nm at an excitation wavelength of 280 nm, and the slit width was maintained at 5 nm. Ultrapure water was used as a blank solution.

### Physical and chemical stability evaluation of nanomicelles

For the thermal stability of nanomicelles, PAI-CAP and PAN-CAP solutions (1 mg/ml) were heated at different temperatures (60, 70, 80, and 90 °C) for 30 min in the dark. For the storage stability, the samples were stored at 4 °C for 0, 7, 14, 21, and 28 days, respectively. Besides that, 0 to 200 mM of NaCl, which covers the range of ionic concentrations in gastrointestinal fluids (the ionic strength of gastric fluid and intestinal fluid is 100 and 140 mM, respectively), were added to evaluate the ionic stability of the nanomicelles [32]. Meantime, pH stability was investigated using a pH of 4.0 to 9.0. The particle size, polydispersity index (PDI), and ζ-potential of nanomicelles were measured by a particle size analyzer (Zetasizer ZEN 3700, Malvern Instruments Ltd., Worcestershire, UK) at 25 °C.

### Stability evaluation of nanomicelles during in vitro simulated digestion

The stability of nanomicelles during gastric and intestinal digestion was referred to INFOGEST 2.0 [33]. The simulated gastric fluid (SGF) and the simulated intestinal fluid (SIF) were prepared and incubated at 37 °C, respectively. The SGF contained 2,000 U/ml of pepsin and different concentrations of electrolyte solutions including 6.9 mM KCl, 0.9 mM KH_2_PO_4_, 25 mM NaHCO_3_, 47.2 mM NaCl, 0.12 mM MgCl_2_(H_2_O)_6_, 0.5 mM (NH_4_)_2_CO_3_, 15.6 mM HCl, and 0.15 mM CaCl_2_(H_2_O)_2_. The SIF contained 100 U/ml trypsin, 10 mM bile salts, and different concentrations of electrolyte solutions including 6.8 mM KCl, 0.8 mM KH_2_PO_4_, 85 mM NaHCO_3_, 38.4 mM NaCl, 0.33 mM MgCl_2_(H_2_O)_6_, 8.4 mM HCl, and 0.6 mM CaCl_2_(H_2_O)_2_. PAI-CAP and PAN-CAP powders (100 mg) were added into 10 ml of SGF and SIF, which were then adjusted to pH 3.0 and pH 7.0, respectively. Approximately 100 μl of the sample was withdrawn at 30, 60, 90, and 120 min, and an equal volume of prewarmed SGF or SIF was replaced. The withdrawn samples were centrifuged (10,000*g*, 10 min), and 300 μl of acetonitrile was added to the supernatant, which was subsequently analyzed by HPLC. The CAP retention was calculated using Eq. 4.

$$\text{CAP}\;\text{retention}\;(\;\%\;)=\frac{CAP\;\text{retained in the supernatant}}{CAP\;\text{content in simulated digestion fluid}}$$ (4)

### Mucus permeation study using Transwell permeable support system

The Transwell 24-well permeable support system (Corning Inc., MA, USA) was used to assess the mucus permeation of nanomicelles [23]. Porcine intestinal mucus was collected and purified based on the method described by de Sousa et al. [34]. First, 50 mg of porcine intestinal mucus was spread on the Transwell insert to form a mucus barrier. Then, 200 μl of PAI-CAP or PAN-CAP solution was added to the apical chamber and 600 μl of PBS buffer (0.01 M, pH 7.2 to 7.4) was added to the basolateral chamber. The plate was incubated at 37 °C. At 1, 2, 4, 6, and 8 h, 100 μl of the sample was withdrawn from the basolateral chamber, while an equal volume of PBS buffer (0.01 M, pH 7.2 to 7.4) was added. The withdrawn samples were mixed with 300 μl of acetonitrile and then centrifuged (8,000*g*, 5 min). The content of CAP in the supernatant was analyzed by HPLC, and the amount of permeated was calculated according to Eq. 5.

$$\begin{array}{c} \text{Amount of permeated}\;\left(\%\right)=\\ \frac{CAP\;\text{content in the basolateral chamber}\;at\;\text{different time points}}{\text{Initial}\;CAP\;\text{content in apical chamber}} \end{array}$$ (5)

### Trajectory tracking of nanomicelles in mucus

MPT technique was used to track the movement behavior of nanomicelles in intestinal mucus [35]. Nanomicelles were labeled using FITC via covalent reaction and marked as PAI-FITC and PAN-FITC [23]. Before the experiment, approximately 50 mg of porcine intestinal mucus was placed in a confocal dish and equilibrated for 30 min at 37 °C. Then, 10 μl of PAI-FITC and PAN-FITC (1 mg/ml) was added to the mucus and incubated at 37 °C for 2 h. The movement of the nanomicelles was observed by using an inverted super-resolution fluorescence microscope (Multi-SIM, Nikon, Tokyo, Japan) equipped with a 100× oil objective lens and a camera, and 20-s video was captured at a time resolution of 60 ms. The ensemble mean squared displacement (MSD) and effective diffusivity (*D*_eff_) were calculated via Eqs. 6 and 7, respectively. The motion trajectories of at least 100 nanomicelles were analyzed for each sample.

$$\text{MSD}={\left[x\left(t+\tau \right)-x\left(t\right)\right]}^{2}+{\left[y\left(t+\tau \right)-y\left(t\right)\right]}^{2}$$ (6)

$$D_{\text{eff}}=\frac{MSD}{4\tau }$$ (7)

where *x* and *y* represent the coordinates of nanomicelles at a certain time, and τ represents the time scale.

### Visualization of mucus penetration of nanomicelles

The 3D distribution of nanomicelles in the mucus layer was investigated by CLSM [36]. The animal experiments were carried out with approval from the Animal Care and Ethics Committee of the China Agricultural University (approval number: AW41703202-4-1). Male Sprague-Dawley rats (170 to 200 g) were sacrificed and their jejunum (~2.5 cm) was removed. The jejunum was tied at both ends and injected with 0.2 ml of PAI-FITC or PAN-FITC (1 mg/ml). The jejunum loop was then incubated in 5 ml of Krebs–Ringer buffer at 37 °C and 100 rpm for 1 h. After incubation, the nanomicelle solution was discarded and the jejunum loop was cut longitudinally to expose the mucus. Subsequently, 20 μl of Alexa Fluor 555–WGA dye solution (10 μg/ml) was added to stain the mucus for 30 min and the mucus-exposed part of jejunum was observed using a 2-photon laser confocal microscope (A1R MP, Nikon, Tokyo, Japan).

### Cellular uptake studies on Caco-2 cells

Caco-2 cells were maintained in DMEM containing 15% (v/v) FBS, 1% (v/v) l-glutamine, 1% (v/v) nonessential amino acids, 100 U/ml penicillin, and 100 μg/ml streptomycin at 37 °C in 5% CO_2_ and 90% relative humidity. To directly observe the cellular uptake of nanomicelles, NR dye was used to prepare NR-loaded nanomicelles, which were labeled as PAI-NR and PAN-NR, respectively. Caco-2 cells were seeded on 24-well plates. After being cultured for 48 h until the confluence was above 80%, the cells were treated with PAI-NR, PAN-NR, and NR solutions (NR: 1 μg) for 1 and 4 h. Then, the cells were washed with PBS and incubated in 4% paraformaldehyde for 20 min. Subsequently, F-actin was stained with 1× Phalloidin–iFluor 488 dye and the nuclei were stained with DAPI. The cells were observed and imaged using CLSM (AXIOobserze.Z1, Zeiss, Oberkochen, Germany).

To further explore cellular uptake mechanisms, Caco-2 cells were preincubated at 37 °C for 0.5 h with different inhibitors including chlorpromazine (10 μg/ml), indomethacin (50 μM), colchicine (10 μg/ml), and sodium azide (10 mM) for clathrin-, caveolae-, and macropinocytosis-mediated endocytosis and energy-dependent cellular uptake, respectively. After washing with PBS, the cells were treated with PAI-CAP, PAN-CAP, and CAP solutions (CAP: 200 μg/ml) containing the above inhibitors at 37 °C for another 2 h. Moreover, the energy-dependent cellular uptake was also verified by preincubating Caco-2 cells at 4 °C for 0.5 h and then incubating cells with sample solutions at 4 °C for another 2 h. After that, the cells were washed with PBS and disrupted with cell tissue lysate buffer. The amount of CAP in the lysate was detected by HPLC. The cell without incubation with inhibitors was used as a blank group, and the endocytosis rate was calculated according to Eq. 8.

$$\begin{array}{c} \text{Endocytosis rate}\;\left(\%\right)=\\ \frac{CAP\;\text{content of cellular uptake in the sample group}}{CAP\;\text{content of cellular uptake in the control group}}\times 100 \end{array}$$ (8)

### Intracellular transport studies

Different inhibitors including brefeldin A (10 μg/ml), monensin (50 μM), and bafilomycin A1 (10 μg/ml) were used to study the intracellular transport of nanomicelles. To investigate the effect of intracellular transport inhibitors on endocytosis, Caco-2 cells were preincubated with different inhibitors at 37 °C for 0.5 h, followed by replacement with CAP, PAI-CAP, and PAN-CAP solutions (CAP: 200 μg/ml) for an additional 2 h at 37 °C. To investigate the inhibitors’ effect on exocytosis, Caco-2 cells were preincubated with sample solutions at 37 °C for 2 h. Then, the sample solutions were replaced by different inhibitors and incubated at 37 °C for another 2 h. After that, the cells were disrupted and the amount of CAP in the lysate was detected by HPLC.

### Permeability of nanomicelles on Caco-2 cell monolayer

The Caco-2 cell monolayer was constructed using the Transwell 12-well permeable support system (Corning Inc., MA, USA) with seeding at a density of 3 × 10^5^ cells per Transwell insert. The growth medium was changed for the first time after 6 to 16 h of culture and every other day thereafter. After 21 days of culture, the transepithelial electrical resistance (TEER) value of Caco-2 cell monolayer measured by a cell resistance meter (Millicell ERS-2, Millipore, MA, USA) exceeded 800 Ω/cm^2^, making it suitable for subsequent studies.

The Caco-2 cell monolayer was equilibrated in preheated HBSS at 37 °C for 30 min. Then, HBSS in the apical chamber was replaced by CAP, PAI-CAP, and PAN-CAP solutions (CAP: 200 μg/ml) and incubated at 37 °C. After incubation for 2, 4, 6, and 8 h, 100 μl of the sample was withdrawn from the basolateral chamber while an equal volume of HBSS was added. Meantime, the TEER values were determined at each time point. The withdrawn samples were mixed with 300 μl of acetonitrile and then centrifuged (8,000*g*, 5 min). The percentage of CAP transported across cellular monolayer was analyzed by HPLC.

### Transcytosis pathway of nanomicelles

The Caco-2 cell monolayer was incubated with brefeldin A, monensin, and bafilomycin A1 at 37 °C for 1 h. Then, the cell monolayer was washed with HBSS and treated with CAP, PAI-CAP, and PAN-CAP solutions (CAP: 200 μg/ml). After incubation at 37 °C for 2 h, the CAP content in the basolateral chamber was analyzed by HPLC.

### Evaluation of nanomicelles to overcome P-gp-mediated efflux

The ability of nanomicelles to overcome P-gp-mediated efflux was evaluated by adding verapamil inhibitor. The Caco-2 cell monolayer was preincubated with verapamil (100 μM) at 37 °C for 30 min. After being washed with HBSS, the cell monolayer was treated with CAP, PAI-CAP, and PAN-CAP solutions (CAP: 200 μg/ml) containing the verapamil inhibitor at 37 °C for another 8 h. The CAP content in the basolateral chamber was detected by HPLC, and the apparent permeability coefficient (*P*_app_) was calculated according to Eq. 9.

$$P_{\text{app}}=\frac{\mathrm{dQ}}{\mathrm{dt}}\times \frac{1}{A\times C}$$ (9)

where *dQ*/*dt* is the CAP penetration rate (mg/s), *A* is the diffusion area (cm^2^), and *C* is the initial concentration of CAP (mg/cm^3^).

### Permeation of nanomicelles in rat intestine

The permeability of nanomicelles in different intestinal segments of the small intestine was evaluated using the ex vivo method [37]. Male Sprague-Dawley rats (170 to 200 g) were sacrificed, and different intestinal segments (duodenum, jejunum, ileum) were removed. Then, 0.4 ml of PAI-CAP and PAN-CAP solutions was loaded into different intestinal segments (~4 cm) and tied at both ends. The intestinal loops were incubated in 10 ml of Krebs–Ringer buffer at 37 °C and 100 rpm for 2 h. After incubation, the amount of CAP permeated into the Krebs–Ringer buffer was detected by HPLC and *P*_app_ value was calculated according to Eq. 9.

### Statistical analysis

All experiments were repeated at least 3 times. Data were analyzed by IBM SPSS statistics 26 (IBM, NY, USA) for significant differences (*P* < 0.05) and expressed as mean ± SD. All fitted curves were plotted by Origin 2022b (Origin Lab Corp., MA, USA) software.

## Results

### The characterization of PAI and PAN

**Fig. 1.**
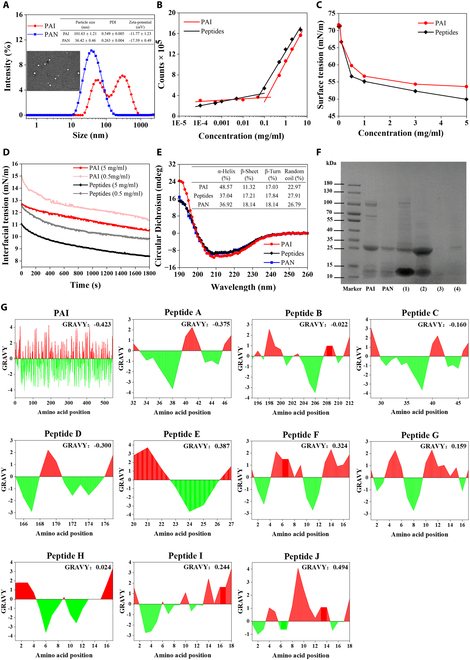
Characterization, separation, and identification of PAN. (A) Particle size distribution of PAI and PAN. The inset shows the TEM image of PAN. Scale bar, 100 nm. (B) Relationship between the maximum fluorescence intensity of NR and protein concentration. (C) Changes in the surface tension of PAI and peptide solutions at different concentrations (0 to 5 mg/ml). (D) Dynamic interfacial tension of PAI and peptide solutions in soybean oil at different concentrations (0.5 and 5 mg/ml). (E) CD spectra of PAI, peptides, and PAN at 190 to 260 nm. The inset shows the percentages of α-helix, β-sheet, β-turn, and random coil. (F) SDS-PAGE of PAI, PAN, and the different fractions collected. Lane 1, markers; lane 2, PAI; lane 3, PAN; lanes 4 to 7, 4 constituent peaks of PAN collected from SEC, recorded as PAN (1), PAN (2), PAN (3), and PAN (4), respectively. (G) Amino acid distribution diagram and peptide hydrophobicity score (GRAVY). Peptides A to E are present in the PA2 chain. Peptides F to K are not found in the database but with a high confidence of de novo results [average local confidence (%) > 50]. The red color and green color in the diagram represent the hydrophobic area and hydrophilic area, respectively.

As shown in Fig. 1A, the average particle size of PAI was 101.63 ± 1.21 nm with a bimodal distribution (PDI > 0.5), while the average particle size of PAN was only 36.42 ± 0.46 nm with a relatively uniform distribution (PDI < 0.3). Meanwhile, a uniform and neatly rounded sphere with a smooth surface and a compact structure was observed, demonstrating the successful formation of self-assembly nanomicelles. The CMC values of PAI and PAN were calculated at the intersection point of 2 fitting curves, 0.13 mg/ml for PAI and 0.062 mg/ml for PAN, respectively (Fig. 1B). The lower CMC value of PAN implied a greater potential to form aggregates with hydrophobic substances, which might be related to unfolding of the structure and exposure of hydrophobic groups during enzymatic hydrolysis. As depicted in Fig. 1C and D, PAN exhibited significantly lower surface tension and oil–water interfacial tension compared to PAI at equivalent concentrations, highlighting the superior ability of PAN to reduce surface tension and interfacial tension, which played a key role in the self-assembly of nanomicelles. This might be attributed to the exposure of their hydrophobic groups and the presence of more carboxyl groups, which were more easily adsorbed in a homogeneous arrangement at the interface, thus increasing the surface activity [38]. The changes in the secondary structure of PAI, peptides, and PAN were represented in Fig. 1E. PAI, peptides, and PAN had 2 major negative troughs at 209 and 222 nm, indicating the existence of α-helix structure. Specifically, PAI displayed a substantial α-helix content of 48.57%, with a relatively lower β-sheet content of 11.32%. After enzymatic hydrolysis, the α-helix content of peptides and PAN decreased to 37.04% and 36.92%, and the β-sheet content increased to 17.12% and 18.14%, respectively, while the β-turn and random coil did not change significantly. This showed that enzymatic hydrolysis could disrupt the α-helix structure and promote the formation of β-sheet structure.

### Separation and characterization of PAN

The typical hydrophobic peptides were separated and collected to evaluate the self-assembly characteristics of PAN. The elution profiles and information on the different fractions eluted by SEC are shown in Fig. S1 and Table S1, respectively, with a peak emergence time of 19 to 23 min for PAI and 25 to 29 min for PAN. This indicated that PAN obtained by enzymatic hydrolysis and pH-shifting method has a smaller molecular weight. After the separation by SEC, the molecular weight composition of PAN was further analyzed by SDS-PAGE (Fig. 1F). It showed that the main molecular weights of PAI focused on 25 and 100 kDa, with only minimal distribution observed at 70 kDa and 10 to 15 kDa. However, the main bands of PAN were located at 25 kDa and 10 to 15 kDa, which indicated that enzymatic hydrolysis by trypsin effectively reduced the molecular weights of PAI. When considering the typical compositions in PAN, PAN (1) had a peak emergence time of 15.80 min and the molecular weight was primarily in the range of 10 to 15 kDa. PAN (2) showed a peak emergence time of 27.86 min with molecular weights of 25 kDa and 10 to 15 kDa. Interestingly, PAN (2) had the most identical bands with PAN, suggesting its significant contribution to the principal compositions of PAI following enzymatic hydrolysis. Furthermore, almost no bands were observed for PAN (3) and PAN (4), due to their tiny molecular weights with small amounts.

Furthermore, peptide sequence of PAN (2) was characterized by liquid chromatography–mass spectrometry (LC-MS), where the detailed amino acid composition and molecular weight are shown in Table S2 and Fig. S2. Ten of the most abundant peptides (A to J) were selected for hydrophilicity assessment. Generally, the molecular weights of A to E were 1,614.825, 1,844.894, 2,119.084, 1,427.668, and 1,010.544 Da, respectively. These peptides (A to E) were found to exist on the PA2 chain of PAI, which implied that PA2 had better potential to form nanomicelles after trypsin hydrolysis (Fig. S3) [13,39]. Meanwhile, in order to identify the hydrophilic and hydrophobic regions in the peptide chains, a schematic based on the Kyte and Doolittle algorithm was constructed (Fig. 1G). The cross distribution of hydrophobic and hydrophilic regions indicated the amphiphilicity of the peptides. The grand average of hydropathicity (GRAVY) values for peptides A to E were all greater than the GRAVY value for the PAI itself. This suggested that PA2 exposed the hydrophobic moiety and produced a more hydrophobic polypeptide after being specifically hydrolyzed by trypsin. From the results of the de novo sequencing (peptides F to J), all GRAVY values were positive. It meant that peptides F to J were highly hydrophobic, and their hydrophobic interactions were more favorable to drive the formation of nanomicelles.

### The characterization of PAI-CAP and PAN-CAP

**Fig. 2.**
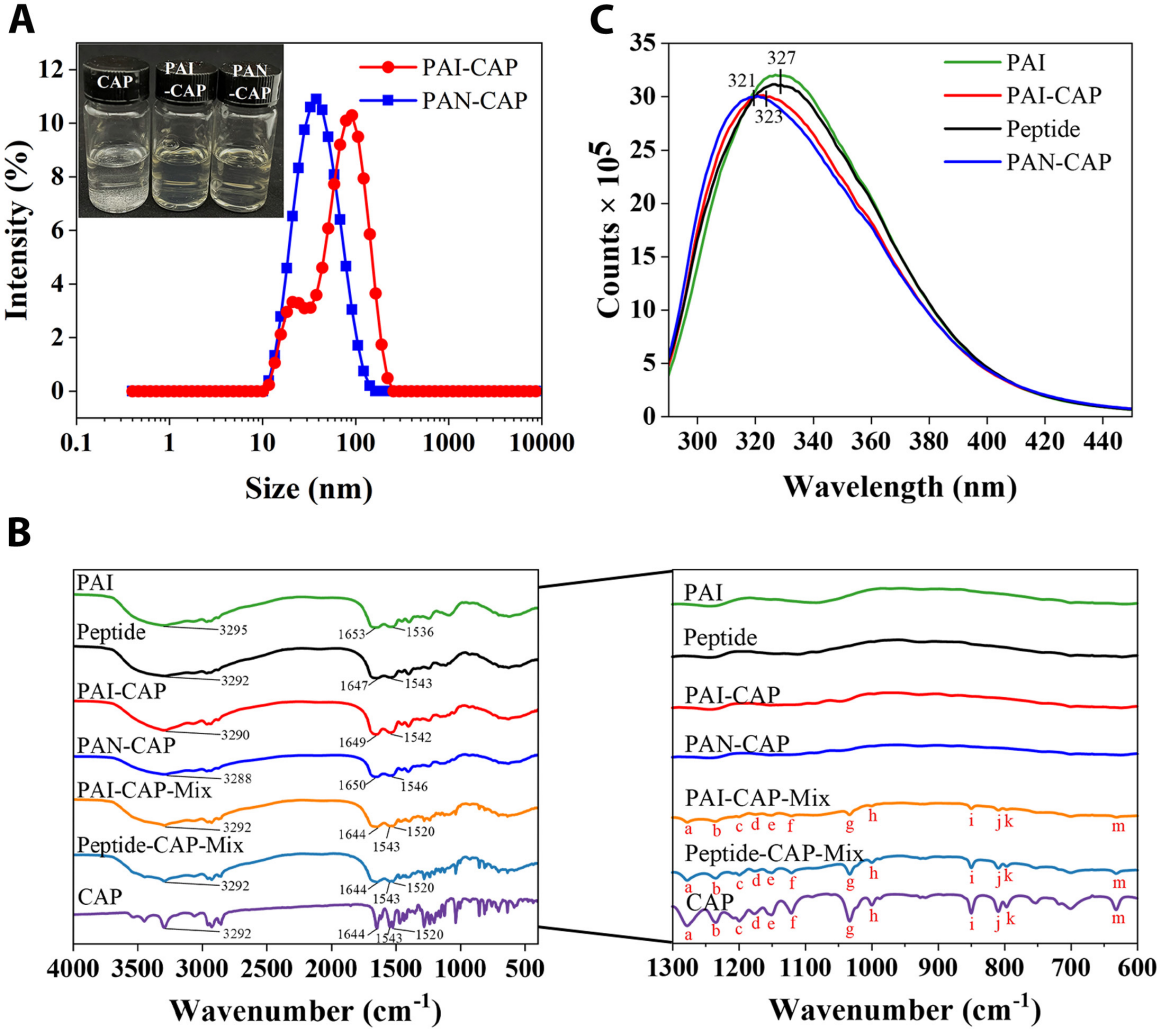
Characterization of PAI-CAP and PAN-CAP. (A) Particle size distribution diagrams for PAI-CAP and PAN-CAP. The inset shows the visual observation. (B) FTIR spectra of CAP, PAI, peptide, encapsulated nanomicelles (PAI-CAP and PAN-CAP), and physically mixed samples (PAI-CAP-Mix and Peptide-CAP-Mix, CAP to PAI/PAP weight ratio = 1:50). Band with significant variation in FT-IR (600 to 1,300 cm^−1^). a to m represent the characteristic peaks of the free CAP, respectively. (C) Intrinsic fluorescence spectra of PAI, peptides, PAI-CAP, and PAN-CAP.

The particle size distribution and appearance of the samples are shown in Fig. 2A, the EE and LA values are displayed in Table S3. As observed from Fig. 2A (inset), PAN-CAP has better water solubility than free CAP. Compared to PAI-CAP, PAN-CAP showed a higher EE value (85.09 ± 0.017%), LA value (20.02 ± 0.433 μg/mg), and smaller particle size (32.83 ± 0.274 nm). This might be related to the more hydrophobic groups exposed after enzymatic hydrolysis of PAI. Moreover, PAI-CAP showed a distinct double peak, while PAN-CAP exhibited a single peak distribution, which indicated that PAN-CAP had a better homogeneity. FTIR spectrum was used to explore whether CAP was encapsulated in PAN successfully (Fig. 2B). It was found that most of the characteristic peaks of CAP vanished in the spectra of PAI-CAP and PAN-CAP, but were still detectable in the spectra of the physical mixtures (PAI-CAP-Mix and PAN-CAP-Mix), confirming the successful encapsulation of CAP in PAN [40]. Additionally, the O–H stretching vibrational absorption peak of the peptide shifted from 3,292 to 3,288 cm^−1^ after encapsulation of CAP, suggesting that hydrogen bonding between CAP and the peptide occurred [41]. Meanwhile, the C=O stretching vibrational and N–H bending vibrational absorption peaks of the peptide shifted from 1,647 and 1,543 cm^−1^ to 1,650 and 1,546 cm^−1^, respectively, implying that hydrophobic interactions also existed between CAP and the peptide. The interaction between CAP and peptide was also studied by intrinsic fluorescence (Fig. 2C). A decrease in fluorescence intensity was observed in peptide compared to PAI, a similar phenomenon to previous studies, probably due to the loss of a small fraction of hydrophobic groups during enzymatic hydrolysis and pH shifting [42,43]. Besides, encapsulation of CAP in PAI and PAN further led to a significant reduction in fluorescence intensity and a blue shift in the maximum emission wavelength. This suggested that the intrinsic fluorescence of PAI/PAN was quenched by CAP, leading the internal fluorophores into a more hydrophobic environment, and also implied that CAP bound to the peptide through hydrophobic interactions. Compared with PAI-CAP (from 327 nm shifted to 323 nm), PAN-CAP showed a more significant blue shift (from 327 nm shifted to 321 nm), indicating that PAN is more prone to bind to CAP and form tighter and denser nanomicelles. This result further validated that PAI exposed hydrophobic groups after enzymatic hydrolysis, thereby enhancing its affinity for CAP binding through hydrophobic interactions.

### Stability of nanomicelles

**Fig. 3.**
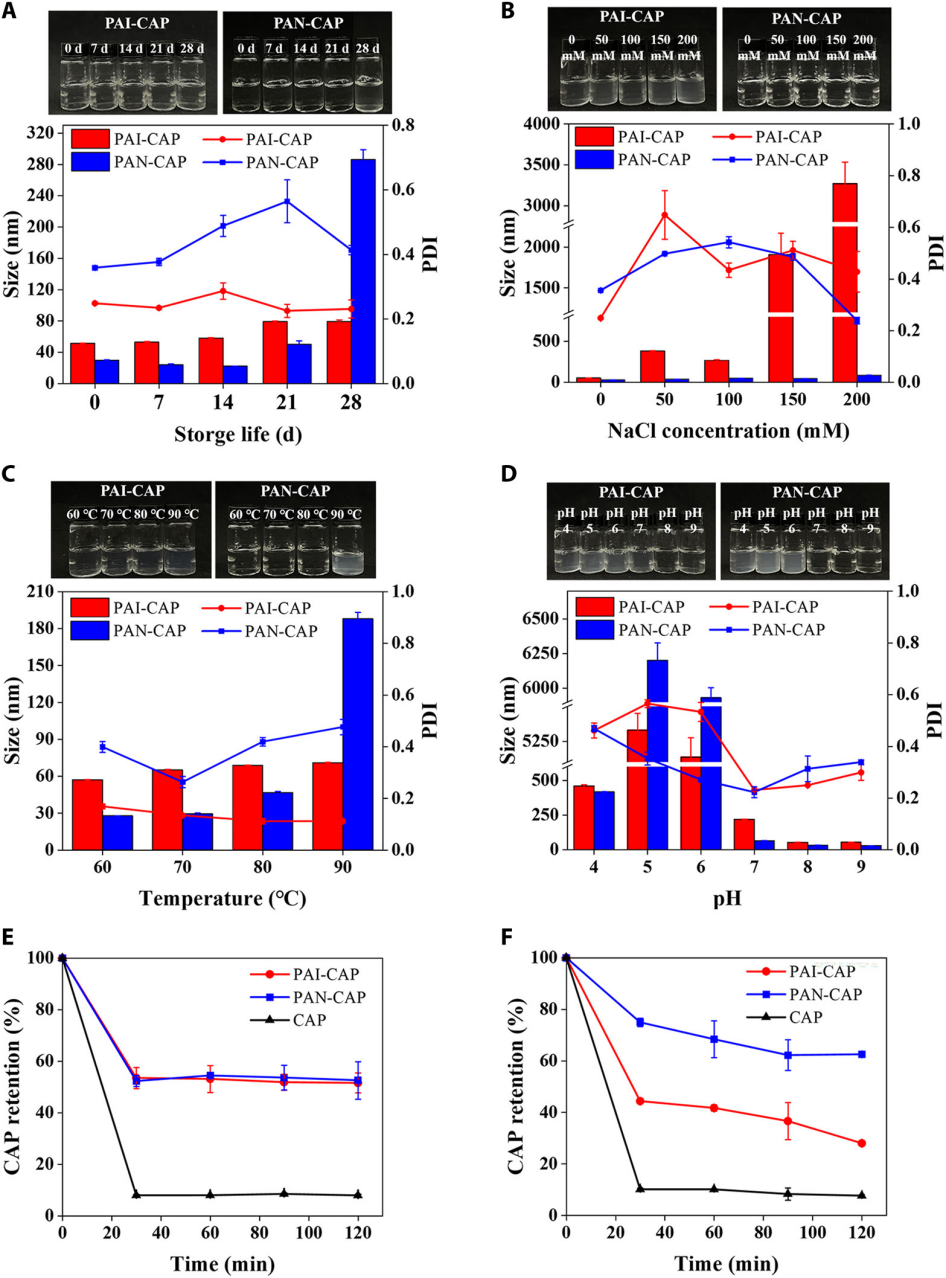
Stability of PAI-CAP and PAN-CAP*.* (A) Storage stability. (B) Ionic stability. (C) Temperature stability. (D) pH stability. (E) Gastric stability. (F) Intestinal stability. The inset shows the corresponding visual appearance. The particle size and PDI measurements were maintained at pH 7.5 and 25 °C.

The stability of nanomicelles under different physicochemical conditions was investigated (Fig. 3). The storage stability experiment (Fig. 3A) demonstrated that the particle size of PAN-CAP was basically constant during 0 to 21 days but suddenly increased to 286.1 nm at 28 days. This indicated that PAN-CAP was more stable during the 21-day storage period and the increase in particle size at 28 days was mainly due to the Brownian motion and the effect of gravity, leading to the occurrence of aggregation of nanomicelles. Whereas, PAI-CAP did not change remarkably in particle size, PDI, and sample appearance during 0 to 28 days, indicating that PAI-CAP was stable during the 28-day storage period. The ionic stability experiment (Fig. 3B) showed that PAN-CAP remained stable at 0 to 200 mM NaCl. The high stability of PAN-CAP to salt ions might be related to its dense structure, which reduced the effect of electrostatic shielding of the salt ions, thus preventing nanomicelle aggregation [43]. However, the particle size of PAI-CAP sharply increased to 382.7 nm and the PDI increased to 0.65 at 50 mM NaCl. Moreover, this encapsulation seemed to be disrupted and formed the precipitate when the NaCl concentration reached more than 150 mM. The temperature stability experiment (Fig. 3C) showed that PAN-CAP remained relatively stable at 60 to 80 °C since the particle size increased slowly. However, when the temperature was increased to 90 °C, PAN-CAP became turbid, where the particle size increased to 187.97 nm, indicating that aggregation and destabilization had occurred. In contrast, PAI-CAP remained consistently stable at 60 to 90 °C, with little change in its particle size remaining around 60 nm. The pH stability experiment (Fig. 3D) showed that the particle size and turbidity of both PAI-CAP and PAN-CAP increased significantly under acidic conditions (pH 4.0 to 6.0). This was mainly due to the fact that the proximity of this pH range to the isoelectric point of PAI (pI = 4.8) led to a near-zero net charge on the surface of the protein, which further reduced interparticle repulsion and resulted in destabilization. Conversely, the particle size of PAI-CAP remained around 53 nm and that of PAN-CAP around 32 nm under alkaline conditions (pH 7.0 to 9.0). It meant that both PAI-CAP and PAN-CAP had good stability under conditions similar to intestinal pH. This could be attributed to the increase in the negative charge on the surface of nanomicelles with increasing pH, leading to an increase in the electrostatic repulsion between the nanomicelles. To sum up, PAI and PAN are indeed comparable in the physicochemical stability. PAI is naturally stable and can maintain stability for 28 days under 4 °C storage conditions in the absence of external interference. Similarly, PAN performs well too, since it can remain stable for 21 days and shows distinct advantages in ionic stability. These stability advantages support further processing and application of PAN in the future.

The gastric stability and intestinal stability were evaluated by measuring the retention of CAP in PAI-CAP and PAN-CAP nanomicelles during simulated in vitro digestion (Fig. 3E and F). It was observed from Fig. 3E that only about 10% free CAP was left in the supernatant after a 2-h gastric digestion process due to its hydrophobicity. However, PAI-CAP and PAN-CAP could largely promote the retention of CAP up to 50% during gastric digestion. This was mainly attributed to a certain inherent resistance of pea albumin to digestive enzymes, allowing some nanomicelles to remain intact and preserve CAP within their hydrophobic core [18,19]. Meanwhile, it could be seen that PAI and PAN had the same CAP retention rate after 2 h of gastric digestion. This was mainly because neither of them underwent pre-enzymatic hydrolysis by pepsin during the preparation, resulting in a similar degree of destruction during gastric digestion. It was also surprising that PAN played a critical role in protecting CAP during intestinal digestion, as more than 65% of CAP was still embedded in those nanomicelles (Fig. 3F). The protective effect of PAN was significantly greater than that of PAI, which retained only approximately 25% of CAP. It might be related to the better anti-intestinal digestive properties of the PAN, which were mainly derived from the fact that it had fewer trypsin enzymatic cleavage sites after pre-enzymatic hydrolysis by trypsin during the preparation process.

### Mucus permeation ability of nanomicelles

**Fig. 4.**
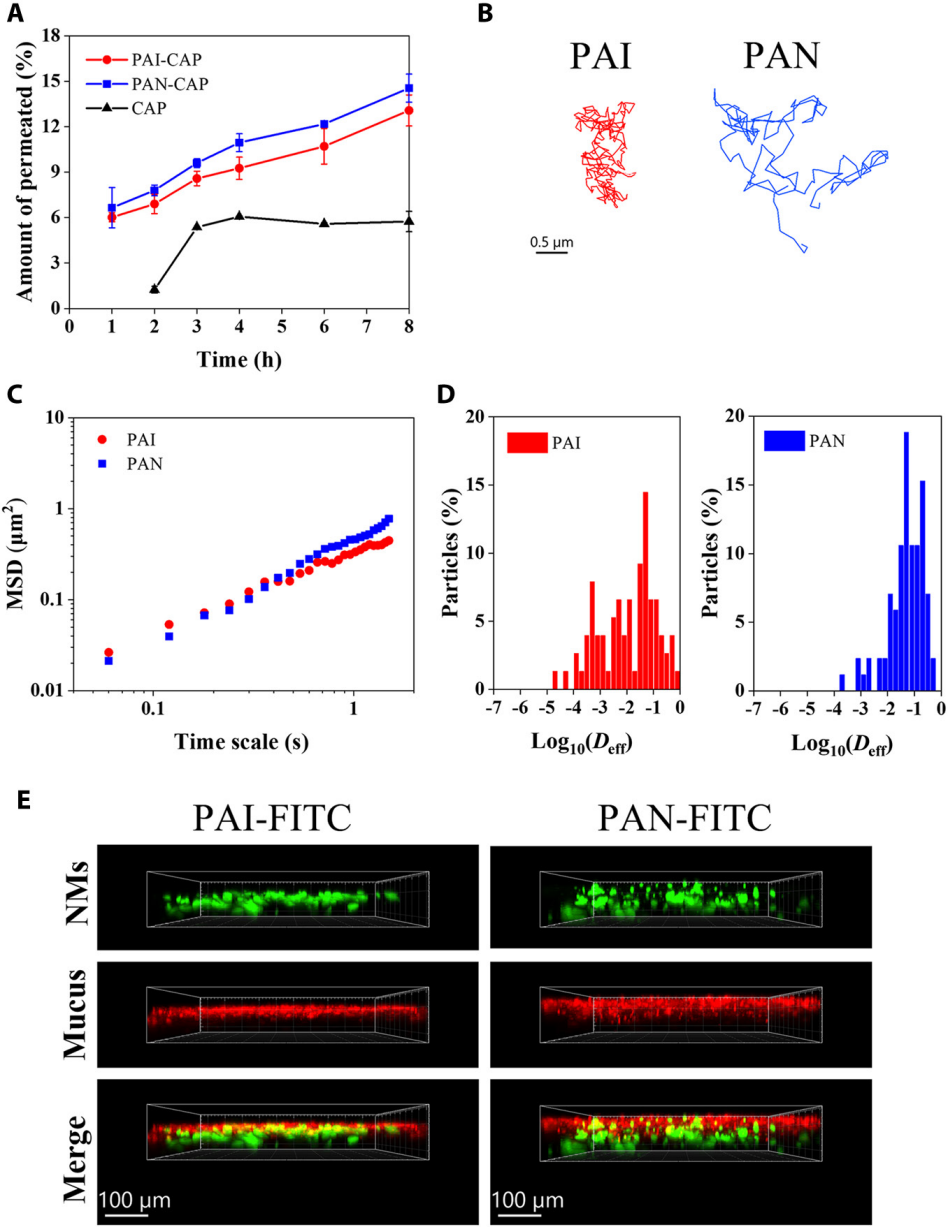
Mucus penetration of PAI and PAN nanomicelles. (A) Permeated amount of CAP, PAI-CAP, and PAN-CAP through mucus layer in a Transwell permeable support system. (B) Representative trajectories of PAI and PAN nanomicelles in rat intestinal mucus at time scales of 10 s. Red, PAI; blue, PAN. (C) Ensemble mean squared displacement (MSD) of PAI and PAN nanomicelles as a function of time scale. (D) Distribution of the logarithm of the effective diffusivity (*D*_eff_) of PAI and PAN nanomicelles at a time scale of 1 s. (E) 3D distribution of PAI and PAN nanomicelles in rat jejunal mucus. Red, mucus stained with Alexa Fluor 555–WGA; green, FITC-labeled nanomicelles.

The nanomicelles that remain structurally intact after gastrointestinal digestion need to overcome the mucus barrier covering the surface of the small intestine to reach the apical side of intestinal epithelial cells. Therefore, purified porcine intestinal mucus was used in the Transwell permeable support system to evaluate the mucus permeability of PAI-CAP and PAN-CAP, respectively. The amount of permeated nanomicelles was determined by measuring the CAP content in the basolateral chamber within 0 to 8 h, and the results are shown in Fig. 4A. It was detected that the amount of permeated PAI-CAP and PAN-CAP across the mucus increased gradually with time prolonged and was always higher than that of the free CAP. When the time extended to 8 h, the amount of permeated PAI-CAP and PAN-CAP reached 13% and 15%, which were 2.2 and 2.5 times higher than that of free CAP, respectively. This indicated that PAI and PAN could significantly improve the mucus permeability of CAP. Moreover, the amount of permeated PAN-CAP was consistently higher than that of PAI-CAP within 8 h, suggesting superior mucus permeability of PAN-CAP.

To further investigate the movement behavior of nanomicelles in intestinal mucus, MPT technology was applied. The representative trajectories of nanomicelles in intestinal mucus are displayed in Fig. 4B. Compared with PAI, PAN nanomicelles moved freely in a broader area, indicating that PAN nanomicelles have a better diffusion ability in the intestinal mucus. Then, the time-dependent displacement of the nanomicelles was further evaluated by calculating the MSD value that was positively correlated with the mucus permeability of nanomicelles (Fig. 4C). The MSD value of PAN was 1.63-fold higher than that of PAI, which again suggested that PAN could penetrate the mucus more efficiently and had a stronger ability to overcome the mucus barrier. Also, the distribution of logarithm of *D*_eff_ was examined and depicted in Fig. 4D. The *D*_eff_ values of PAN were mainly concentrated between 0.01 and 1 μm^2^/s, while those of PAI had a large distribution between 0.0001 and 1 μm^2^/s. This proved that the PAN had a faster diffusion rate in the intestinal mucus.

To verify whether the nanomicelles with better mucus permeability have a deeper distribution in the mucus, the 3D fluorescence images of the distribution of nanomicelles in mucus were observed (Fig. 4E). Although some of the PAI nanomicelles penetrated the mucus layer, a larger number of them were still trapped in the mucus layer. In contrast, most of the PAN nanomicelles could penetrate through the mucus layer, suggesting that PAN with stronger mucus permeability have a deeper distribution in the mucus than PAI.

### Cellular uptake of nanomicelles

**Fig. 5.**
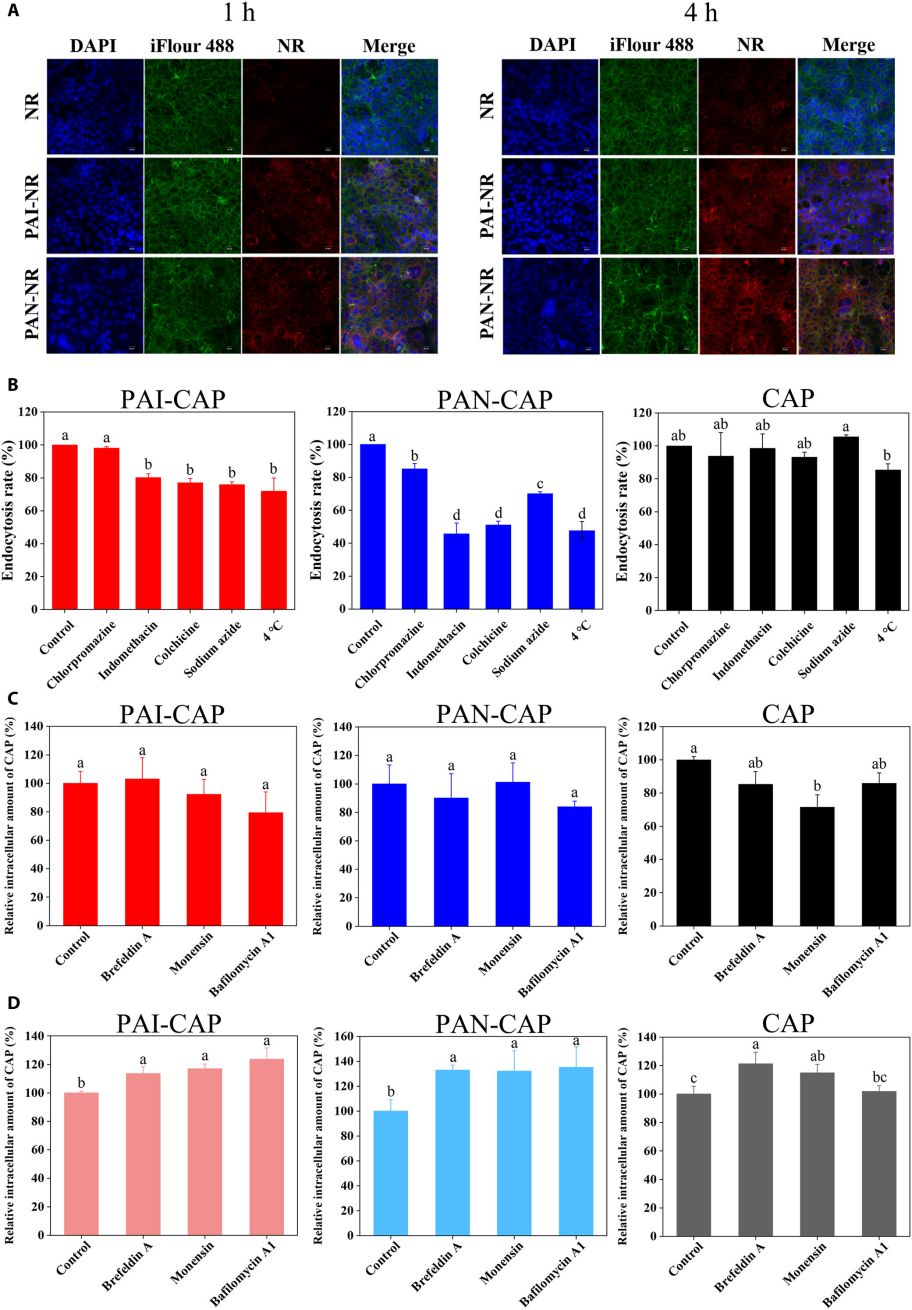
Cellular uptake and intracellular transport of PAI-CAP, PAN-CAP, and CAP. (A) CLSM images of Caco-2 cells incubated with PAI-NR, PAN-NR, and NR for 1 and 4 h. Blue, nucleus stained with DAPI; green, F-actin stained with iFluor 488; red, NR-loaded nanomicelles or free NR. (B) Cellular uptake mechanisms of PAI-CAP, PAN-CAP, and CAP in Caco-2 cells. (C) Effect of intracellular transport inhibitors on endocytosis of PAI-CAP, PAN-CAP, and CAP. (D) Effect of intracellular transport inhibitors on exocytosis of PAI-CAP, PAN-CAP, and CAP.

The cytotoxicity of PAI-CAP, PAN-CAP, and CAP was evaluated before cellular uptake investigation (Fig. S4). Cell viability of 3 groups began to decline significantly (*P* < 0.05) when the CAP concentration was higher than 0.2 mg/ml. Therefore, 0.2 mg/ml was chosen as the CAP dose for the subsequent experiments. The cellular uptake of nanomicelles was characterized by CLSM (Fig. 5A). The red fluorescence around the nucleus in free NR was very weak, while the distribution and intensity of red fluorescence in PAI-NR and PAN-NR were significantly increased, indicating that nanomicelles could promote the uptake of hydrophobic substances by Caco-2 cells. With the extension of incubation time from 1 to 4 h, the fluorescence in free NR did not change significantly, indicating that the cellular uptake of NR had reached saturation. This might be because NR was taken up by cells through a passive transport pathway that NR was no longer taken up by cells when intra- and extracellular NR concentrations reached equilibrium. On the contrary, the fluorescence intensities in PAI-NR and PAN-NR were significantly enhanced with the time prolonging, which might be related to the ability of nanomicelles to alter the endocytosis pathway of free molecules to achieve cellular uptake through energy-dependent active transport. Meanwhile, the red fluorescence intensity of PAN-NR was always higher than that of PAI-NR, indicating that PAN-NR was more easily taken up by Caco-2 cells.

To further determine the cellular uptake mechanisms of the nanomicelles, the cells were treated with different endocytosis inhibitors. The endocytosis inhibitors used had no effect on cell viability (Fig. S5). As shown in Fig. 5B, endocytosis rates of both PAI-CAP and PAN-CAP were significantly (*P* < 0.05) reduced in the presence of sodium azide or at 4 °C, revealing that PAI-CAP and PAN-CAP were taken up via an energy-dependent process. For PAI-CAP, endocytosis rate was significantly inhibited (*P* < 0.05) in the presence of indomethacin and colchicine, suggesting that PAI-CAP was taken up by caveolae- and macropinocytosis-mediated endocytosis. For PAN-CAP, endocytosis rate decreased to 85%, 45%, and 51% in the presence of chlorpromazine, indomethacin, and colchicine, respectively. This indicated that the uptake of PAN-CAP was primarily mediated by caveolae and macropinocytosis, with an additional contribution from clathrin. In addition, none of the inhibitors had any effect on the endocytosis of free CAP, suggesting that the cellular uptake of CAP molecules did not require energy involvement and may be related to passive transport.

### Intracellular transport of nanomicelles

To explore the intracellular transport pathways of nanomicelles, 3 inhibitors (brefeldin A, monensin, and bafilomycin A1) were used to block specific intracellular transport pathways. Brefeldin A specifically suppresses positive transport between the endoplasmic reticulum (ER) and Golgi, monensin inhibits transport from Golgi to plasma membrane, while bafilomycin A1 inhibits endosomal acidification and retrograde membrane transport at the ER/Golgi complexes [44]. These 3 inhibitors had no effect on cell viability (Fig. S6). As illustrated in Fig. 5C, 3 inhibitors showed negligible inhibition effect on the endocytosis of PAI-CAP and PAN-CAP, indicating that ER, Golgi, and endoplasmic acidification did not affect the endocytosis of nanomicelles. Endocytosis of free CAP was only inhibited in the monensin, suggesting that the transport process from Golgi to plasma membrane may influence the endocytosis of free CAP. Inhibitors blocking intracellular transport pathways may also have an impact on exocytosis, as shown in Fig. 5D. For PAI-CAP and PAN-CAP, 3 inhibitors all significantly increased (*P* < 0.05) the retention of CAP in the cells, indicating that endoplasmic acidification, the transport from ER to Golgi, and the transport from Golgi to the plasma membrane were all involved in exocytosis, whereas, for free CAP, the retention of CAP in the cells increased only in the presence of brefeldin A and bafilomycin A1, suggesting that only the transport from ER to Golgi and the transport from Golgi to the plasma membrane were involved in exocytosis of free CAP.

### Transcellular transport of nanomicelles

**Fig. 6.**
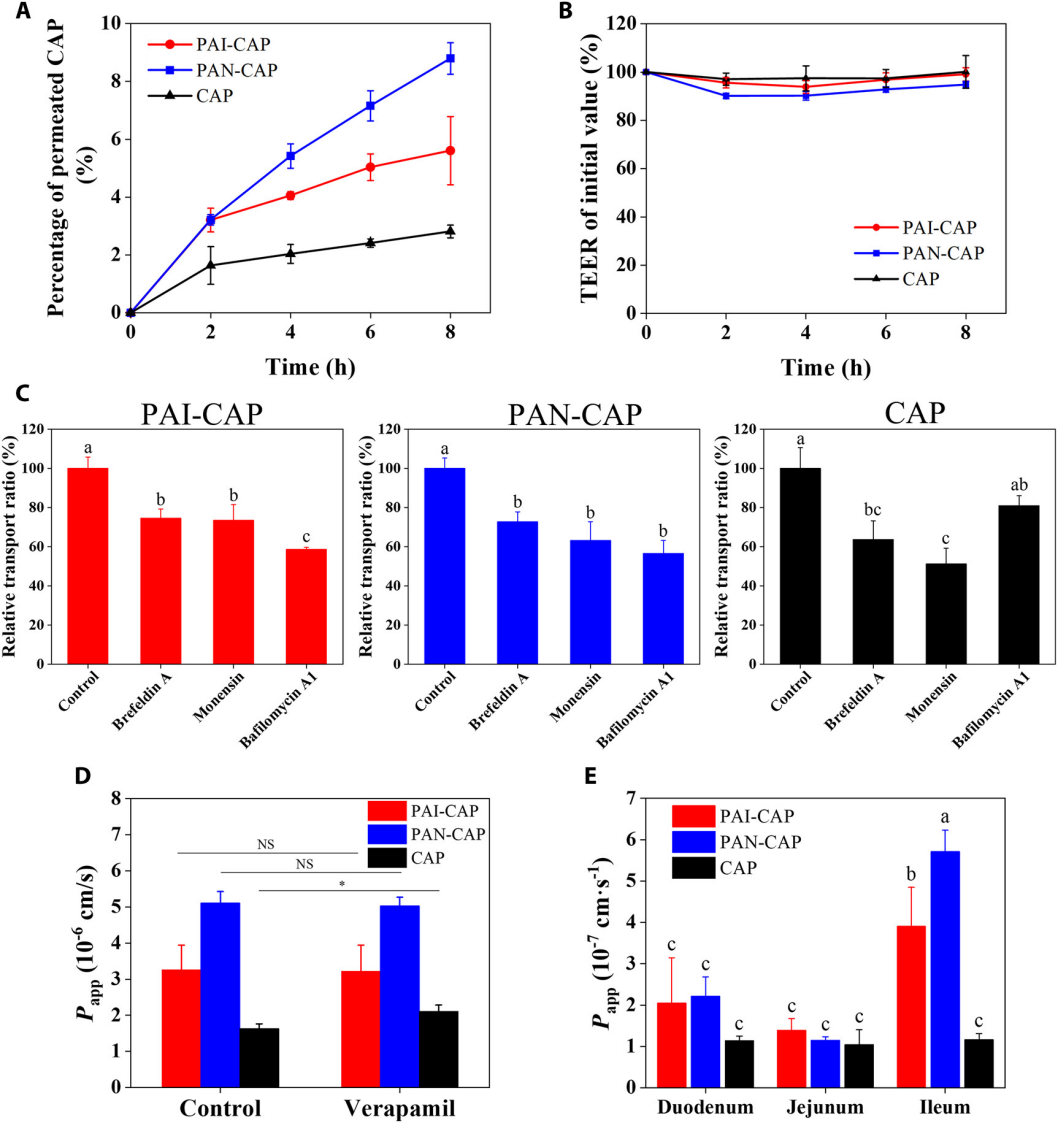
Transcellular transport and intestinal permeability of PAI-CAP, PAN-CAP, and CAP. (A) Permeated percentage of PAI-CAP, PAN-CAP, and CAP through Caco-2 monolayer. (B) Changes of TEER value of Caco-2 monolayers incubated with PAI-CAP, PAN-CAP, and CAP within 8 h. (C) Effect of inhibitors on transcytosis of PAI-CAP, PAN-CAP, and CAP. (D) Effect of verapamil inhibitor on the apparent permeability coefficient (*P*_app_) of PAI-CAP, PAN-CAP, and CAP. (E) Apparent permeability coefficient (*P*_app_) of PAI-CAP, PAN-CAP, and CAP in the duodenum, jejunum, and ileum.

The transepithelial permeability of nanomicelles was studied using the Caco-2 cell monolayer. The permeated percentage of PAI-CAP, PAN-CAP, and CAP through Caco-2 monolayer is shown in Fig. 6A. PAN-CAP demonstrated the highest permeated percentage within 2 to 8 h, which was 1.57- and 3.14-fold higher than that of PAI-CAP and free CAP at 8 h. This suggested that nanomicelles could enhance the transport of CAP across Caco-2 cell monolayer. Additionally, PAN-CAP was easier to transport through Caco-2 monolayers than PAI-CAP, which may be attributed to the smaller particle size of PAN-CAP. Meanwhile, the TEER values of the Caco-2 cell monolayer were monitored during permeability studies (Fig. 6B). The TEER values of PAI-CAP, PAN-CAP, and CAP did not change significantly within 8 h, revealing that none of them transport across the Caco-2 cell monolayer via the paracellular pathway.

Furthermore, the transcytosis pathway of nanomicelles was investigated using various inhibitors (Fig. 6C). The relative transport ratios of PAI-CAP, PAN-CAP, and CAP across Caco-2 monolayer were significantly decreased (*P* < 0.05) with the addition of brefeldin A and monensin, indicating that the transport from ER to Golgi and from Golgi to plasma membrane was involved. In addition, PAI-CAP and PAN-CAP showed reduced relative transport ratios with the addition of bafilomycin A1, while free CAP did not change markedly. This suggested that the transport of PAI-CAP and PAN-CAP involved endosomal acidification, whereas the transport of free CAP may not. These findings corresponded with the results of intracellular transport mechanism studies (Fig. 5C and D).

To verify whether the nanomicelles could effectively mitigate the P-glycoprotein (P-gp) efflux effect, the inhibitor of P-gp efflux action (verapamil) was used to preincubate the Caco-2 cell monolayer. Figure 6D demonstrated the *P*_app_ values of PAI-CAP, PAN-CAP, and CAP in the presence or absence of verapamil. It was observed that the *P*_app_ value of free CAP was significantly increased (*P* < 0.05) in the presence of verapamil, but there was no significant change in *P*_app_ values of PAI-CAP and PAN-CAP. This suggested that the transcellular transport of free CAP was inhibited by the P-gp efflux effect, however, nanomicelles could overcome the P-gp efflux effect on the transport of CAP and improved its transcellular transport ratio.

### Intestinal permeability evaluation of nanomicelles

To further confirm that nanomicelles could enhance the intestinal permeability of free CAP, the transports of PAI-CAP, PAN-CAP, and CAP across different intestinal tracts were studied via ex vivo method. As shown in Fig. 6E, the permeability of free CAP in different parts of the small intestine was similar, and the *P*_app_ values were all about 1 × 10^−7^ cm s^-1^. The *P*_app_ values of PAI-CAP and PAN-CAP in duodenum and jejunum were not significantly different from that of free CAP, but the *P*_app_ values of PAI-CAP and PAN-CAP in ileum were 3.4 and 4.92 times that of free CAP, respectively. This revealed that nanomicelles could significantly improve the intestinal permeability of free CAP, which may be attributed to its improved mucus permeability and transepithelial transport capacity. In addition, both PAI-CAP and PAN-CAP had the highest *P*_app_ values in the ileum, suggesting that the ileum may be the main absorption site of CAP delivered by nanomicelles. This was possibly due to the fact that the thickness of the mucus layer in the ileum was smaller than that in the duodenum and jejunum, which was conducive to the penetration of nanomicelles [45].

## Discussion

Oral nanodelivery carriers are extensively designed for the delivery of drugs and bioactive compounds, aiming to enhance their bioavailability. However, prior to effective absorption, these oral nanodelivery carriers have to sequentially overcome multiple physical and chemical barriers, including gastrointestinal pH conditions, enzymatic degradation, mucus barrier, and intestinal epithelial barrier. Hence, utilizing nanocarriers endowed with excellent gastrointestinal stability, mucus permeability, and transintestinal epithelial transport capacity for delivery can be more effective. In this study, PANs with these multiple functions were fabricated, which can be used as efficient oral delivery carriers, especially for strongly hydrophobic bioactives such as CAP. CAP is relatively stable under acidic and alkaline conditions at 25 °C, but its thermal and light stability are poor. The main problem that currently limits the application of CAP is its poor water solubility (approximately 10.3 mg/l at 25 °C), which leads to its poor intestinal permeability and consequently reduces its bioavailability [46]. Therefore, encapsulation of CAP using PANs, which possess excellent gastrointestinal stability, mucus permeability, and intestinal epithelial transport capacity, is an effective strategy to improve its intestinal permeability.

**Fig. 7.**
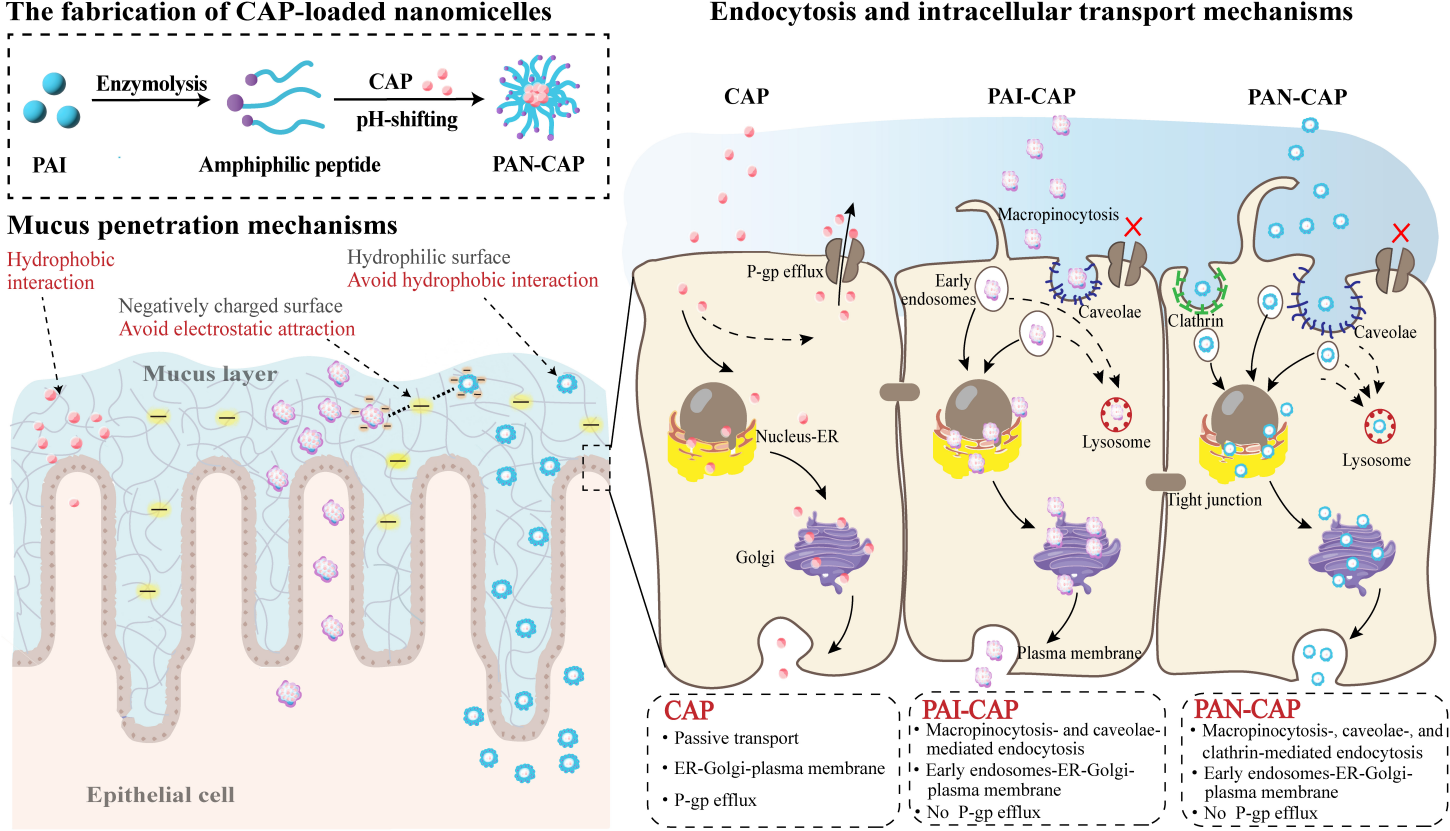
Mucus penetration and intestinal absorption mechanisms of PAI-CAP, PAN-CAP, and CAP.

The fabrication process of nanomicelles, along with their mucus penetration mechanism and intestinal epithelial cell absorption mechanism, is clearly illustrated in Fig. **7**. PAI was first partially hydrolyzed by trypsin to generate amphiphilic peptides. These amphiphilic peptides were then self-assembled into nanomicelles under the pH-shifting process. The self-assembly mechanism was further explored. It was found that the amphiphilic peptides exhibited lower CMC value (0.062 mg/ml), lower surface tension and dynamic interfacial tension values, and higher content of β-sheet structure, which played a key role in the self-assembly process. Meanwhile, the peptide sequencing results verified that the 10 most abundant amphiphilic peptides were all strongly hydrophobic, driving them to form self-assembled nanomicelles through hydrophobic interactions. This also implied the potential to be a good carrier for hydrophobic bioactive compounds. Therefore, CAP was used as a hydrophobic drug model and embedded in the hydrophobic core of nanomicelles for the follow-up study.

PAN-CAP first undergoes a harsh gastrointestinal environment after oral administration, so its gastrointestinal stability was assessed by in vitro simulated digestion. The results showed that PAN was able to protect CAP from rapid release during gastrointestinal digestion, exhibiting a certain degree of gastrointestinal digestion resistance. This protective effect was mainly due to the reduction of enzymatic cleavage sites by pre-enzymatic hydrolysis of PAI using human gut digestive enzymes, which largely weakened the enzymatic hydrolysis effect during in vitro simulated digestion. Thus, the structure of nanomicelles could be better maintained and CAP located in the hydrophobic core of PAN was protected from being rapidly released.

After being subjected to the harsh gastrointestinal environment, PAN-CAP faces the subsequent barrier of permeating the intestinal mucus layer to reach the apical side of the intestinal epithelium. For free CAP, it is difficult to penetrate the mucus due to its strong hydrophobicity. When CAP was encapsulated by PAN, the mucus permeability of CAP was significantly improved. MPT and CLSM experiments showed that PAN has excellent diffusion ability in intestinal mucus and can achieve deeper distribution in the mucus. It was attributed to the fact that PAN had a particle size smaller than the average diameter of the mucus mesh (100 nm) as well as a negatively charged and hydrophilic surface, which avoided electrostatic and hydrophobic interactions with the mucus, thus facilitating easy penetration across the reticulated mucus layer [12,47].

When a small portion of free CAP managed to overcome the mucus barrier and reach the apical membrane of epithelial cells, it primarily relied on passive transport to enter the cells, followed by intracellular transport via the ER–Golgi–plasma membrane pathway. Additionally, due to the presence of P-gp efflux, the transcellular transport rate of free CAP was further reduced, contributing to poor intestinal permeability. When CAP was encapsulated by PAN, its cellular uptake and transcellular transport rate were significantly increased, which can be attributed to changes in the endocytosis pathway. PAN-CAP relied on energy-dependent active transport rather than passive transport for cellular entry. Specifically, it entered cells utilizing macropinocytosis, caveolae, and clathrin-mediated endocytosis pathways. Meantime, PAN-CAP displayed the ability to overcome the P-gp efflux, which can be attributed to 2 aspects. First, the size of PAN-CAP (32.83 ± 0.27 nm) was larger than the molecular size of P-gp, which was about 160 Å long and 45 × 65 Å wide with a 9- to 25-Å extracellular size (1 Å = 0.1 nm) [48]. This size disparity was not conducive for P-gp to pump PAN-CAP out of the cell. Second, it has been reported that P-gp possesses the binding sites of ATP and hydrophobic drugs [49]. When the bound ATP is hydrolyzed by ATPase, it can release energy, which can be utilized by P-gp to pump the bound hydrophobic drugs out of the cell. By encapsulating CAP within the hydrophobic core of PAN, the direct binding of P-gp to CAP was effectively blocked, consequently inhibiting P-gp-mediated CAP efflux. Following cellular uptake, PAN-CAP undergoes intracellular transport via the endosomal acidification–ER–Golgi–plasma membrane pathway for intracellular transport. It has been reported that the caveolae-mediated endocytosis pathway has the ability to avoid lysosomal degradation. During transient interaction with endosomes, the caveosome retains its integrity without disassembling the cavity shell, thus bypassing the lysosomes and contributing to efficient perinuclear trafficking into nucleus, Golgi, and ER [50]. Furthermore, ex vivo intestinal tissue permeation experiments demonstrated that PAN-CAP significantly increased the permeability of CAP in the ileum, as a comprehensive reflection of improvement in mucus permeability and transcellular transport rate of CAP by PAN. In conclusion, PAN shows promise as an oral nanodrug delivery system capable of effectively overcoming the harsh gastrointestinal environment and mucus barrier. This system enhances the intestinal absorption of hydrophobic bioactive substances.

## Data Availability

The data that support the findings of this study are available from the corresponding author upon reasonable request.

## References

[1] Jacob J, Haponiuk JT, Thomas S, Gopi S. Biopolymer based nanomaterials in drug delivery systems: A review, materials today. Chemistry. 2018;9:43–55.

[2] Sung YK, Kim SW. Recent advances in polymeric drug delivery systems. Biomater Res. 2020;24(1):12. 32537239 10.1186/s40824-020-00190-7 PMC7285724

[3] Tong XQ, Pan WH, Su T, Zhang MY, Dong W, Qi XL. Recent advances in natural polymer-based drug delivery systems. React Funct Polym. 2020;148:104501.

[4] Guan T, Zhang Z, Li X, Cui S, McClements DJ, Wu X, Chen L, Long J, Jiao A, Qiu C, et al. Preparation, characteristics, and advantages of plant protein-based bioactive molecule delivery systems. Food Secur. 2022;11(11):1562. 10.3390/foods11111562 PMC9180007 35681312

[5] Zu MH, Ma Y, Cannup B, Xie DC, Jung YJ, Zhang JM, Yang CH, Gao F, Merlin D, Xiao B. Oral delivery of natural active small molecules by polymeric nanoparticles for the treatment of inflammatory bowel diseases. Adv Drug Deliv Rev. 2021;176:113887. 34314785 10.1016/j.addr.2021.113887

[6] Chapa-Villarreal FA, Miller M, Rodriguez-Cruz JJ, Pérez-Carlos D, Peppas NA. Self-assembled block copolymer biomaterials for oral delivery of protein therapeutics. Biomaterials. 2023;300:122191. 37295223 10.1016/j.biomaterials.2023.122191

[7] Yildiz HM, CA MK, Marsac PJ, Carrier RL. Size selectivity of intestinal mucus to diffusing particulates is dependent on surface chemistry and exposure to lipids. J Drug Target. 2015;23(7-8):768–774. 26453172 10.3109/1061186X.2015.1086359 PMC4874559

[8] Wang Q, Zhao Y, Guan L, Zhang Y, Dang Q, Dong P, Li J, Liang X. Preparation of astaxanthin-loaded DNA/chitosan nanoparticles for improved cellular uptake and antioxidation capability. Food Chem. 2017;227:9–15. 28274463 10.1016/j.foodchem.2017.01.081

[9] Liang TS, Zhang ZT, Jing P. Black rice anthocyanins embedded in self-assembled chitosan/chondroitin sulfate nanoparticles enhance apoptosis in HCT-116 cells. Food Chem. 2019;301:125280. 31377624 10.1016/j.foodchem.2019.125280

[10] Tang CH. Nanostructured soy proteins: Fabrication and applications as delivery systems for bioactives (a review). Food Hydrocoll. 2019;91:92–116.

[11] Cone RA. Barrier properties of mucus. Adv Drug Deliv Rev. 2009;61(2):75–85. 19135107 10.1016/j.addr.2008.09.008

[12] Zhang X, Cheng HB, Dong W, Zhang MX, Liu QY, Wang XH, Guan J, Wu HY, Mao SR. Design and intestinal mucus penetration mechanism of core-shell nanocomplex. J Control Release. 2018;272:29–38. 29305112 10.1016/j.jconrel.2017.12.034

[13] Higgins TJV, Beach LR, Spencer D, Chandler PM, Randall PJ, Blagrove RJ, Kortt AA, Guthrie RE. cDNA and protein-sequence of a major pea seed albumin (PA 2-Mr-approximately-26 000). Plant Mol Biol. 1987;8(1):37–45. 24302522 10.1007/BF00016432

[14] Lu ZX, He JF, Zhang YC, Bing DJ. Composition, physicochemical properties of pea protein and its application in functional foods. Crit Rev Food Sci Nutr. 2020;60(15):2593–2605. 31429319 10.1080/10408398.2019.1651248

[15] Yang S, Li X, Hua Y, Chen Y, Kong X, Zhang C. Selective complex coacervation of pea whey proteins with chitosan to purify main 2s albumins. J Agric Food Chem. 2020;68(6):1698–1706. 31986048 10.1021/acs.jafc.9b06311

[16] Stone AK, Karalash A, Tyler RT, Warkentin TD, Nickerson MT. Functional attributes of pea protein isolates prepared using different extraction methods and cultivars. Food Res Int. 2015;76(Pt 1):31–38.

[17] Lu BY, Quillien L, Popineau Y. Foaming and emulsifying properties of pea albumin fractions and partial characterisation of surface-active components. J Sci Food Agric. 2000;80(13):1964–1972.

[18] Pilar Utrilla M, Jesus Peinado M, Ruiz R, Rodriguez-Nogales A, Algieri F, Elena Rodriguez-Cabezas M, Clemente A, Galvez J, Rubio LA. Pea (*Pisum sativum* L.) seed albumin extracts show anti-inflammatory effect in the DSS model of mouse colitis. Mol Nutr Food Res. 2015;59(4):807–819. 25626675 10.1002/mnfr.201400630

[19] Park SJ, Kim TW, Baik B-K. Relationship between proportion and composition of albumins, and in vitro protein digestibility of raw and cooked pea seeds (*Pisum sativum* L.). J Sci Food Agric. 2010;90(10):1719–1725. 20564440 10.1002/jsfa.4007

[20] Akharume FU, Aluko RE, Adedeji AA. Modification of plant proteins for improved functionality: A review. Compr Rev Food Sci Food Saf. 2021;20(1):198–224. 33393195 10.1111/1541-4337.12688

[21] Wouters AGB, Rombouts I, Fierens E, Brijs K, Delcour JA. Relevance of the functional properties of enzymatic plant protein hydrolysates in food systems. Compr Rev Food Sci Food Saf. 2016;15(4):786–800. 33401841 10.1111/1541-4337.12209

[22] Liu B, Liu B, Wang R, Li Y. Alpha-lactalbumin self-assembled nanoparticles with various morphologies, stiffnesses, and sizes as Pickering stabilizers for oil-in-water emulsions and delivery of curcumin. J Agric Food Chem. 2021;69(8):2485–2492. 33555192 10.1021/acs.jafc.0c06263

[23] Yuan D, Zhou F, Niu Z, Shen P, Zhao M. Formation of mucus-permeable nanoparticles from soy protein isolate by partial enzymatic hydrolysis coupled with thermal and pH-shifting treatment. Food Chem. 2023;398:133851. 35963217 10.1016/j.foodchem.2022.133851

[24] Lv Y, Chen L, Liu F, Xu F, Zhong F. Improvement of the encapsulation capacity and emulsifying properties of soy protein isolate through controlled enzymatic hydrolysis. Food Hydrocoll. 2023;138:108444.

[25] Liu S, Sun N, Ren KY, Tan XB, Li LX, Wang Z, Dai SC, Tong XH, Wang H, Jiang LZ. Utilization of self-assembled soy protein nanoparticles as carriers for natural pigments: Examining non-interaction mechanisms and stability. Food Hydrocoll. 2024;148(Pt B).

[26] Chen L, Lv Y, Zhong F. Enhancing bioavailability of soy protein isolate (SPI) nanoparticles through limited enzymatic hydrolysis: Modulating structural properties for improved digestion and absorption. Food Hydrocoll. 2024;147(Pt B):109397.

[27] Rubio LA, Perez A, Ruiz R, Angeles Guzman M, Aranda-Olmedo I, Clemente A. Characterization of pea (*Pisum sativum*) seed protein fractions. J Sci Food Agric. 2014;94(2):280–287. 23744804 10.1002/jsfa.6250

[28] Nielsen PM, Petersen D, Dambmann C. Improved method for determining food protein degree of hydrolysis. J Food Sci. 2001;66(5):642–646.

[29] Mallick S, Song SJ, Bae Y, Choi JS. Self-assembled nanoparticles composed of glycol chitosan-dequalinium for mitochondria-targeted drug delivery. Int J Biol Macromol. 2019;132:451–460. 30930268 10.1016/j.ijbiomac.2019.03.215

[30] Hu Y, Bao C, Li D, You L, Du Y, Liu B, Li X, Ren F, Li Y. The construction of enzymolyzed alpha-lactalbumin based micellar nanoassemblies for encapsulating various kinds of hydrophobic bioactive compounds. Food Funct. 2019;10(12):8263–8272. 31720654 10.1039/c9fo02035g

[31] Wu X, Xu N, Cheng C, McClements DJ, Chen X, Zou L, Liu W. Encapsulation of hydrophobic capsaicin within the aqueous phase of water-in-oil high internal phase emulsions: Controlled release, reduced irritation, and enhanced bioaccessibility. Food Hydrocoll. 2022;123:107184.

[32] Chen J, Zheng J, McClements DJ, Xiao H. Tangeretin-loaded protein nanoparticles fabricated from zein/β-lactoglobulin: Preparation, characterization, and functional performance. Food Chem. 2014;158:466–472. 24731371 10.1016/j.foodchem.2014.03.003

[33] Brodkorb A, Egger L, Alminger M, Alvito P, Assuncao R, Ballance S, Bohn T, Bourlieu-Lacanal C, Boutrou R, Carriere F, et al. INFOGEST static in vitro simulation of gastrointestinal food digestion. Nat Protoc. 2019;14(4):991–1014. 30886367 10.1038/s41596-018-0119-1

[34] de Sousa IP, Cattoz B, Wilcox MD, Griffiths PC, Dalgliesh R, Rogers S, Bernkop-Schnürch A. Nanoparticles decorated with proteolytic enzymes, a promising strategy to overcome the mucus barrier. Eur J Pharm Biopharm. 2015;97(Pt A):257–264. 25661320 10.1016/j.ejpb.2015.01.008

[35] Hu S, Pei X, Duan L, Zhu Z, Liu Y, Chen J, Chen T, Ji P, Wan Q, Wang J. A mussel-inspired film for adhesion to wet buccal tissue and efficient buccal drug delivery. Nat Commun. 2021;12(1):1689. 33727548 10.1038/s41467-021-21989-5 PMC7966365

[36] Fan W, Xia D, Zhu Q, Li X, He S, Zhu C, Guo S, Hovgaard L, Yang M, Gan Y. Functional nanoparticles exploit the bile acid pathway to overcome multiple barriers of the intestinal epithelium for oral insulin delivery. Biomaterials. 2018;151:13–23. 29055774 10.1016/j.biomaterials.2017.10.022

[37] Cheng H, Cui Z, Guo S, Zhang X, Huo Y, Mao S. Mucoadhesive versus mucopenetrating nanoparticles for oral delivery of insulin. Acta Biomater. 2021;135:506–519. 34487859 10.1016/j.actbio.2021.08.046

[38] Rousseau D. Fat crystals and emulsion stability—A review. Food Res Int. 2000;33(1):3–14.

[39] Gressent F, Da Silva P, Eyraud V, Karaki L, Royer C. Pea albumin 1 subunit b (PA1b), a promising bioinsecticide of plant origin. Toxins. 2011;3(12):1502–1517. 22295174 10.3390/toxins3121502 PMC3268454

[40] Zhang X, Wang C, Qi Z, Zhao R, Wang C, Zhang T. Pea protein based nanocarriers for lipophilic polyphenols: Spectroscopic analysis, characterization, chemical stability, antioxidant and molecular docking. Food Res Int. 2022;160:111713. 36076408 10.1016/j.foodres.2022.111713

[41] Dai S, Lian Z, Qi W, Chen Y, Tong X, Tian T, Lyu B, Wang M, Wang H, Jiang L. Non-covalent interaction of soy protein isolate and catechin: Mechanism and effects on protein conformation. Food Chem. 2022;384:132507. 35217462 10.1016/j.foodchem.2022.132507

[42] Braudo EE, Danilenko AN, Guslyannikov PV, Kozhevnikov GO, Artykova GP, Lapteva NA, Vaintraub IA, Sironi E, Duranti M. Comparative effects of limited tryptic hydrolysis on physicochemical and structural features of seed 11S globulins. Int J Biol Macromol. 2006;39(4-5):174–178. 16787660 10.1016/j.ijbiomac.2005.12.006

[43] Yuan D, Zhou F, Shen P, Zhang Y, Lin L, Zhao M. Self-assembled soy protein nanoparticles by partial enzymatic hydrolysis for pH-driven encapsulation and delivery of hydrophobic cargo curcumin. Food Hydrocoll. 2021;120:106759.

[44] Zhang J, Liu D, Zhang M, Sun Y, Zhang X, Guan G, Zhao X, Qiao M, Chen D, Hu H. The cellular uptake mechanism, intracellular transportation, and exocytosis of polyamidoamine dendrimers in multidrug-resistant breast cancer cells. Int J Nanomed. 2016;11:3677–3690. 10.2147/IJN.S106418 PMC4977074 27536106

[45] Paone P, Cani PD. Mucus barrier, mucins and gut microbiota: The expected slimy partners? Gut. 2020;69(12):2232–2243. 32917747 10.1136/gutjnl-2020-322260 PMC7677487

[46] Rezazadeh A, Hamishehkar H, Ehsani A, Ghasempour Z, Moghaddas Kia E. Applications of capsaicin in food industry: Functionality, utilization and stabilization. Crit Rev Food Sci Nutr. 2021;63(19):4009–4025. 34751073 10.1080/10408398.2021.1997904

[47] Lai SK, Wang Y-Y, Hanes J. Mucus-penetrating nanoparticles for drug and gene delivery to mucosal tissues. Adv Drug Deliv Rev. 2009;61(2):158–171. 19133304 10.1016/j.addr.2008.11.002 PMC2667119

[48] Halder J, Pradhan D, Kar B, Ghosh G, Rath G. Nanotherapeutics approaches to overcome P-glycoprotein-mediated multi-drug resistance in cancer. Nanomed Nanotechnol Biol Med. 2022;40:102494. 10.1016/j.nano.2021.102494 34775061

[49] Kaur V, Garg T, Rath G, Goyal AK. Therapeutic potential of nanocarrier for overcoming to P-glycoprotein. J Drug Target. 2014;22(10):859–870. 25101945 10.3109/1061186X.2014.947295

[50] Mazumdar S, Chitkara D, Mittal A. Exploration and insights into the cellular internalization and intracellular fate of amphiphilic polymeric nanocarriers. Acta Pharm Sin B. 2021;11(4):903–924. 33996406 10.1016/j.apsb.2021.02.019 PMC8105776

