# Supplementary material for: Natural Gastrointestinal Stable Pea Albumin Nanomicelles for Capsaicin Delivery and Their Effects for Enhanced Mucus Permeability at Small Intestine
Source: Biomater Res. 2024 Aug 16;28:0065. doi: 10.34133/bmr.0065 (PMC11327615; doi:10.34133/bmr.0065)
Supplement: Supplementary 1 — Figs. S1 to S6 Tables S1 to S3 [file bmr.0065.f1.docx]

**Supplementary Materials**

**Natural Gastrointestinal Stable Pea Albumin Nanomicelles for Capsaicin Delivery and Their Effects for Enhanced Mucus Permeability at Small Intestine**

Yiming Li ^1†^, Mengqi Mao ^1†^, Xin Yuan ^1^, Jiajia Zhao ^1^, Lingjun Ma^1^ , Fang Chen ^1^, Xiaojun Liao ^1^, Xiaosong Hu ^1^, Junfu Ji ^1*^

^1^College of Food Science and Nutritional Engineering, National Engineering Research Center for Fruit and Vegetable Processing, China Agricultural University, Key Lab of Fruit and Vegetable Processing, Ministry of Agriculture and Rural Affairs, Beijing 100083, China.

^*^Address correspondence to: [junfu.ji@cau.edu.cn](mailto:junfu.ji@cau.edu.cn)

^†^These authors contributed equally to this work.

**Materials and Methods**

**Cytotoxicity studies**

The cck-8 assay was used to evaluate the cytotoxicity of CAP, PAI-CAP and PAN-CAP on Caco-2 cells. Caco-2 cells were seeded on 96-well plates (10000 cells per well). After cultured for 24 h, the cells were treated with 10 μL of sample solutions containing different concentrations of CAP for 24 h. Following that, the cells were washed with PBS and incubated with 10 μL of cck-8 reagent for 2 h. The absorbance value at 450 nm was measured. The groups without cells and sample incubation were used as a blank group and a control group, respectively. The cell viability was calculated according to the following equation.

$$Cell viability \left( \% \right)=\frac{A_{sample, 450 nm}-A_{blank, 450 nm}}{A_{control, 450 nm}-A_{blank, 450 nm}}$$

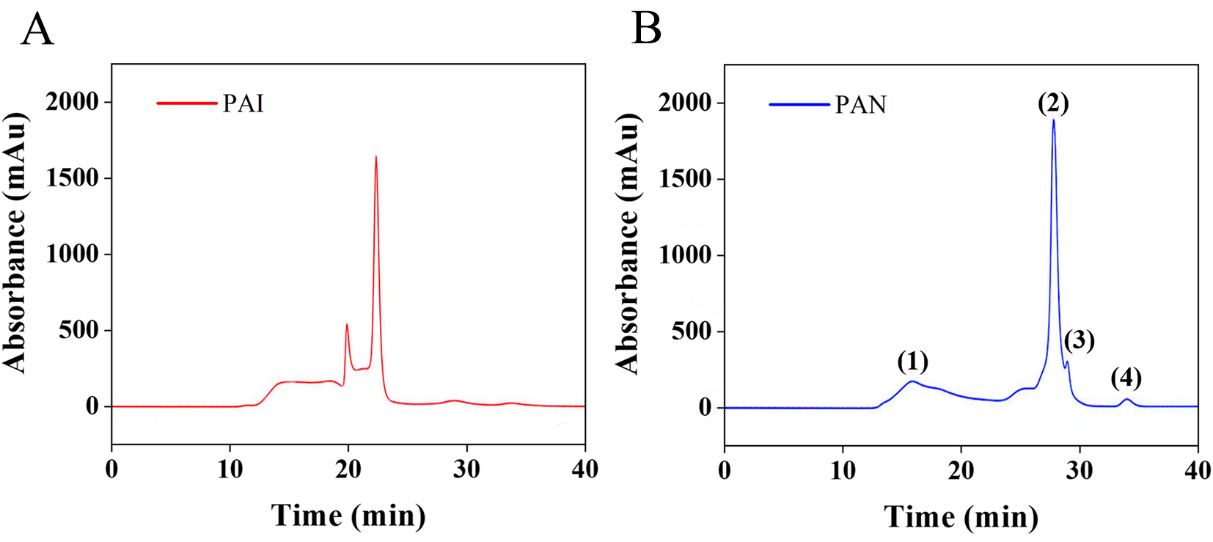


**Figure S1.** SEC elution profile of PAI (A) and PAN (B).


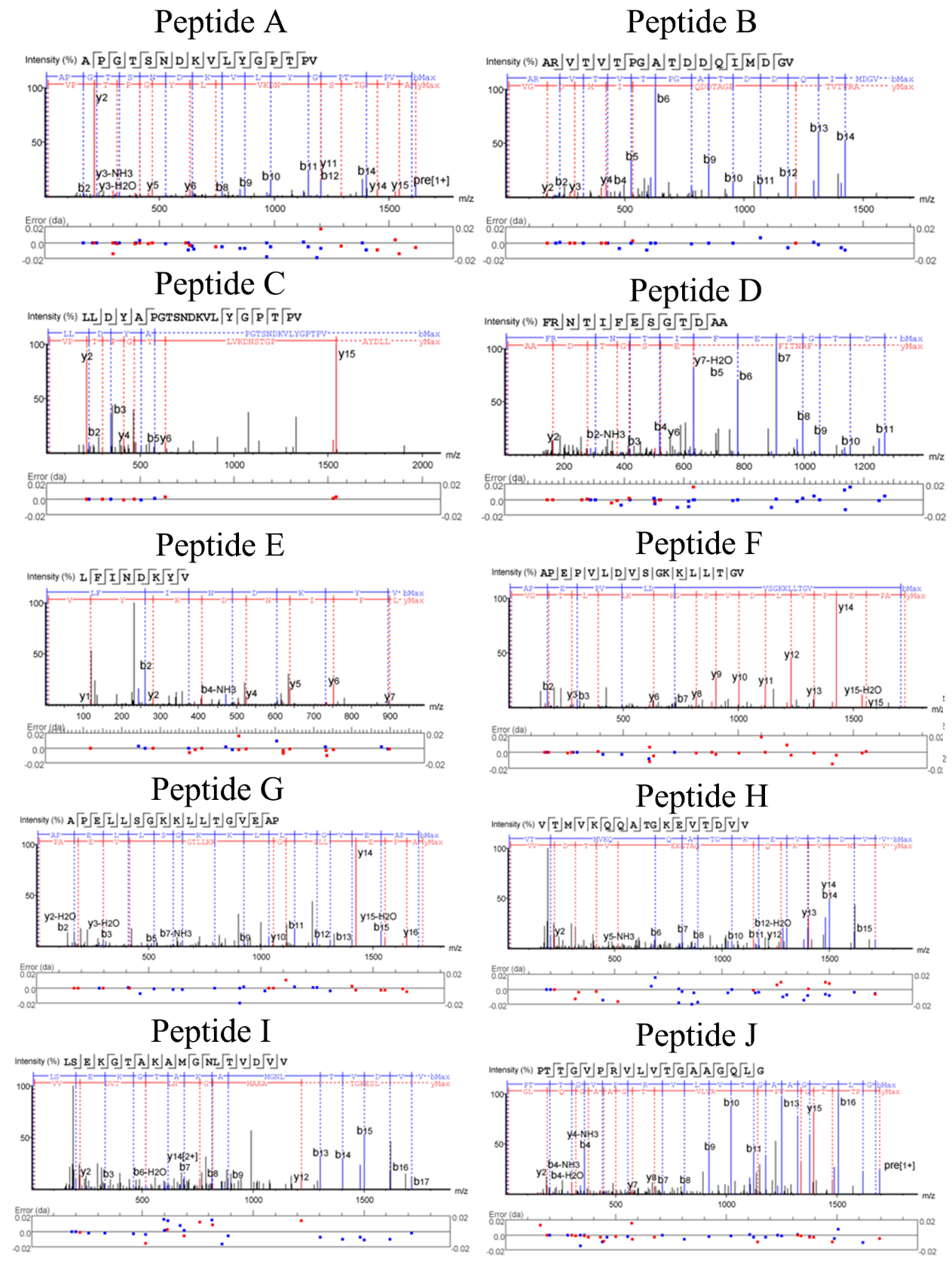


**Figure S2.** Secondary mass spectra of Peptide A-Peptide J.


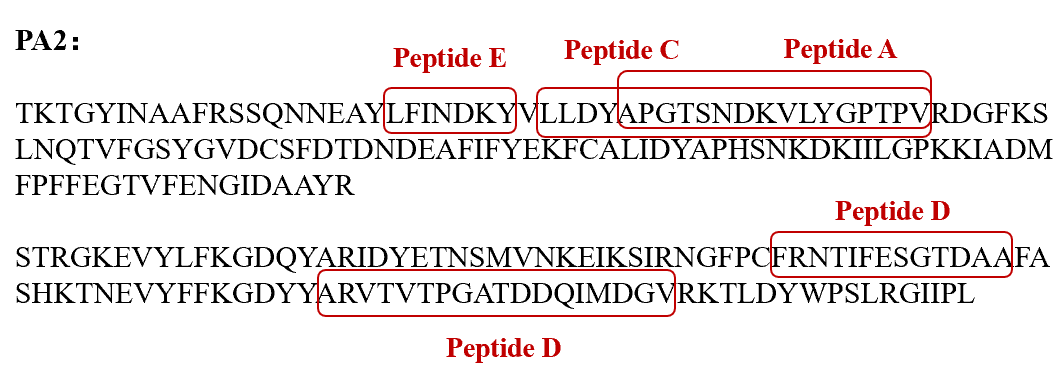


**Figure S3.** Location distribution of Peptide A-Peptide E on the PA2 chain.


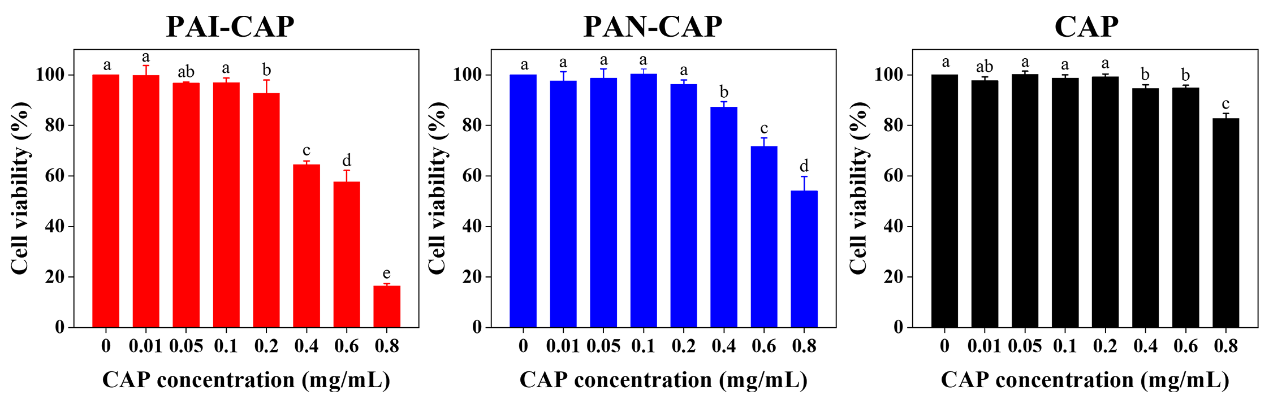


**Figure S4.** Cytotoxicity of PAI-CAP, PAN-CAP and CAP on Caco-2 cells.


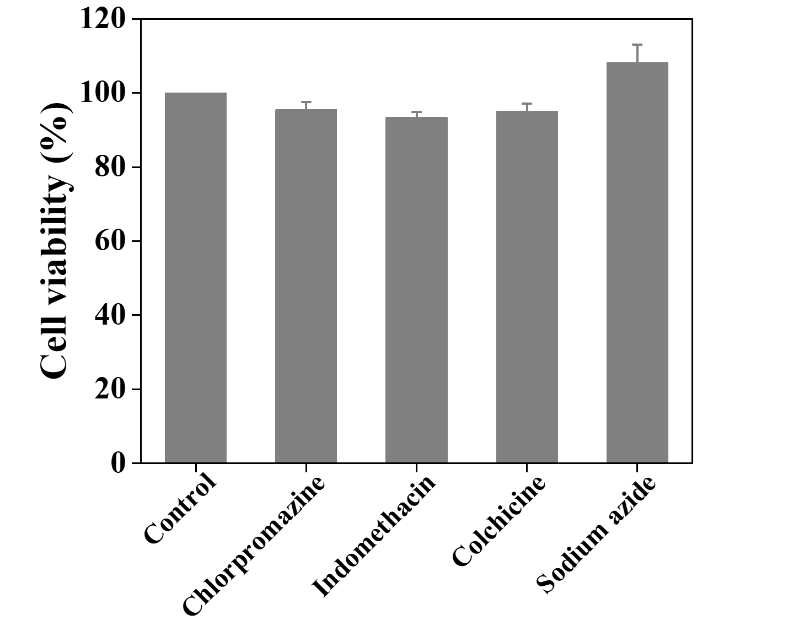


**Figure S5.** Cytotoxicity of various endocytosis inhibitors on Caco-2 cells.


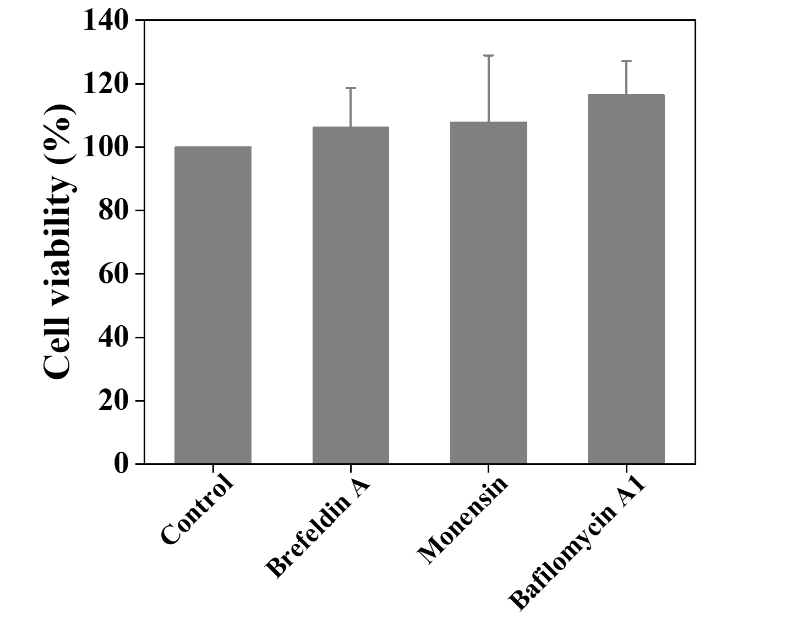


**Figure S6.** Cytotoxicity of various intracellular transport inhibitors on Caco-2 cells.

**Table S1.** Information about the different fractions eluted by SEC.

| Number | Retention time (min) | Peak area | Area percent (%) |
| --- | --- | --- | --- |
| PAN (1) | 15.80 | 13.66 | 0.68 |
| PAN (2) | 27.86 | 1820.35 | 91.03 |
| PAN (3) | 28.98 | 162.82 | 8.14 |
| PAN (4) | 34.01 | 2.85 | 0.14 |

**Table S2.** Identification of purified peptides.

| Peptides | Sequence | Number of amino acids | ppm | m/z | molecular weight (D) |
| --- | --- | --- | --- | --- | --- |
| A | APGTSNDKVLYGPTPV | 16 | 1.8 | 808.4214 | 1614.825 |
| B | ARVTVTPGATDDQIMDGV | 18 | 2 | 923.4560 | 1844.894 |
| C | LLDYAPGTSNDKVLYGPTPV | 20 | 1.3 | 1060.5505 | 2119.084 |
| D | FRNTIFESGTDAA | 13 | 1.5 | 714.8424 | 1427.668 |
| E | LFINDKYV | 8 | 2 | 506.2801 | 1010.544 |
| F | APEPVLDVSGKKLLTGV | 17 | 2.6 | 575.0063 | 1721.993 |
| G | APELLSGKKLLTGVEAP | 17 | 2.6 | 575.0063 | 1721.993 |
| H | VTMVKQQATGKEVTDVV | 17 | 1.6 | 611.6654 | 1831.971 |
| I | LSEKGTAKAMGNLTVDVV | 18 | 1.6 | 611.6654 | 1831.971 |
| J | PTTGVPRVLVTGAAGQLG | 18 | 1.6 | 847.4847 | 1692.952 |

**Table S3.** Particle size, PDI, encapsulation efficiency (EE) and loading amount (LA) of PAI-CAP and PAN-CAP.

|  | Particle size  (nm) | PDI | EE  (%) | LA  (μg/mg nanomicelles) |
| --- | --- | --- | --- | --- |
| PAI-CAP | 66.75 ± 0.233 ^b^ | 0.292 ± 0.007 ^b^ | 74.56 ± 0.016 ^a^ | 16.41 ± 0.064 ^a^ |
| PAN-CAP | 32.83 ± 0.274 ^a^ | 0.229 ± 0.006 ^a^ | 85.09 ± 0.017 ^b^ | 20.02 ± 0.433 ^b^ |

The different lowercase superscript letters in a column indicate a significant difference (*p* < *0.05*).
